# Suppression of the myofibroblastic cancer-associated fibroblast phenotype to enhance anti-PD-1 response

**DOI:** 10.1186/s13046-026-03779-3

**Published:** 2026-07-10

**Authors:** Katherine A. Johnson, Anna E.L. Lippert, Alec Cornelio, Yousef Gadalla, Xingqi Shen, Sean G. Kraus, Philip B. Emmerich, Anna M. Field, Alyssa K. Steimle, Cheri A. Pasch, Huy Q. Dinh, Wei Zhang, Kristina A. Matkowskyj, Dustin A. Deming

**Affiliations:** 1 Division of Hematology, Medical Oncology, and Palliative Care, Department of Medicine, University of Wisconsin School of Medicine and Public Health, University of Wisconsin-Madison, Madison, WI USA; 2 University of Wisconsin Carbone Cancer Center, Madison, WI USA; 3 McArdle Laboratory for Cancer Research, Department of Oncology, University of Wisconsin-Madison, Madison, WI USA; 4 Department of Pathology and Laboratory Medicine, University of Wisconsin– Madison, Madison, WI USA; 5 Department of Laboratory Medicine and Pathology, Mayo Clinic, Rochester, MN USA

**Keywords:** Colorectal cancer, Cancer-associated fibroblasts, Tumor microenvironment, Myocardin, Nilotinib, Anti-PD-1

## Abstract

**Background:**

Cancer-associated fibroblasts (CAFs) are key modulators of tumor growth, the tumor immune context, and treatment response. CAFs take on diverse phenotypes with distinct functions. Myofibroblastic CAFs (myCAFs) regulate extracellular matrix remodeling, while inflammatory CAFs (iCAFs) modulate immune infiltration. Further studies are required to validate biomarkers of CAF phenotypes and to identify means to therapeutically manipulate CAFs.

**Methods:**

Single-cell RNA sequencing analysis was used to identify markers of CAF phenotypes in CRC. CAF markers were validated and compared to clinical characteristics using patient-derived CAF cultures, fixed human CRC samples, and publicly available RNA sequencing data. Therapeutics were prioritized for their potential to inhibit the myCAF phenotype and nilotinib was selected for validation in patient-derived CAFs. Overall changes in CAFs were evaluated via RNA-seq, and results validated with in vitro experiments. Finally, the feasibility of altering CAF phenotypes to affect treatment response was evaluated in vivo*.*

**Results:**

Here we identify disease-specific markers of colorectal CAFs that are functionally distinct, biologically identifiable, correlate with clinically relevant markers of disease, and can be therapeutically altered in vivo. High stromal expression of myCAF markers correlated with reduced infiltration of CD8^+^ T lymphocytes into the epithelial compartment compared to tumors with high stromal expression of iCAF markers. Nilotinib treatment inhibited myogenesis regulators myocardin and myocardin-related transcription factor A, which correlated with reduced expression of myCAF markers and extracellular matrix proteins and decreased tumor size in colorectal cancer-bearing mice when combined with anti-PD-1 therapy. We also identified PD-1 as a potential molecule that activates myocardin and myCAF expression, indicating a potential mechanism for increased response to the combination with anti-PD-1 therapy.

**Conclusions:**

These results highlight the feasibility of targeting CAF functions to improve therapeutic response, and support the investigation of nilotinib and anti-PD-1 therapy clinically.

**Supplementary Information:**

The online version contains supplementary material available at https://doi.org/10.1186/s13046-026-03779-3.

## Background

Colorectal cancer (CRC) remains a leading cause of cancer-related death despite advances in screening [1]. Therapeutic advances continue to be made for patients with metastatic disease, including advances in immunotherapies. Although anti-PD-1 and anti-CTLA-4 immune checkpoint inhibitors have shown significant improvements for those CRC patients with mismatch repair deficiency, this accounts for only 3% of metastatic CRC patients [2–5]. Recent trials have shown that immunotherapy combinations can be beneficial for some mismatch repair proficient CRCs [6–10], however the subpopulation of patients most likely to respond has yet to be identified.

CAFs are of interest both as key regulators of the immune microenvironment and as potential targets for anticancer therapy [11, 12]. Across several cancer types, different CAF subtypes have been identified that carry out distinct functional roles within the tumor microenvironment (TME). In pancreatic cancer, functional subsets of CAFs have been identified and validated, including myofibroblastic CAFs (myCAFs) that function in extracellular matrix remodeling, inflammatory CAFs (iCAFs) that function in immune signaling, and antigen-presenting CAFs (apCAFs), a rare subset of CAFs that express MHC II molecules [13–15]. Several groups have indicated proposed markers for CAF subtypes in CRC using scRNA-seq data [16, 17]. However, these analyses are often focused on the immune cell compartment and are accompanied by little to no validation of fibroblast markers. Several functional studies of CAF subtypes in CRC have aided in understanding the relationship between CAFs and CRC [18, 19]. However, these studies generally utilize a limited panel of markers to distinguish CAFs borrowed from other cancer types and need further validation in CRC specifically. This validation is important as markers for CAF subsets with similar functions have been found to be distinct for different disease sites [20]. However, a focused and comprehensive analysis of CRC-specific CAF subtypes that encapsulates validation of the presence of disease-specific markers on distinct populations of CAFs in the TME and relates these markers back to unique functions of CAF subtypes of consequence to disease biology is lacking.

Here we present a comprehensive interrogation of CRC CAFs with the goal of identifying disease-specific markers and functions. We identify markers related to known functions of CAFs in the TME. Expression of these markers in the stroma of CRC tumors relates to altered immune infiltration. Nilotinib is identified as a potential therapeutic agent able to modulate CAF phenotypes and functions in the TME. Nilotinib treatment reduces expression of myogenesis transcription factors myocardin (*MYOCD*) and myocardin-related transcription factor alpha (*MRTFA*), key regulators of several myCAF-related genes, providing a potential mechanism for its ability to modulate the myCAF phenotype. Combining nilotinib with anti-PD-1 therapy improved response in in vivo models with high stromal infiltration. These data not only provide a connection between markers of CAF subtypes and functions in CRC, but utilize this relationship to identify a means to target pro-tumor functions of CAFs.

## Materials and methods

### Study design

The aim of this study was to identify CRC-specific markers of CAF subtypes and apply those markers to biological samples to investigate and alter CAF functions. To achieve this aim, scRNA-seq data from 23 CRC patients was re-analyzed, then identified markers and the CAF functions associated with them were evaluated on several different sample types, including immunohistochemical assays of over 206 CRC samples from 153 patients, in vitro assays of primary patient- and mouse-derived CAFs, and in vivo experiments on a cohort of 58 mice.

**Fig. 1:**
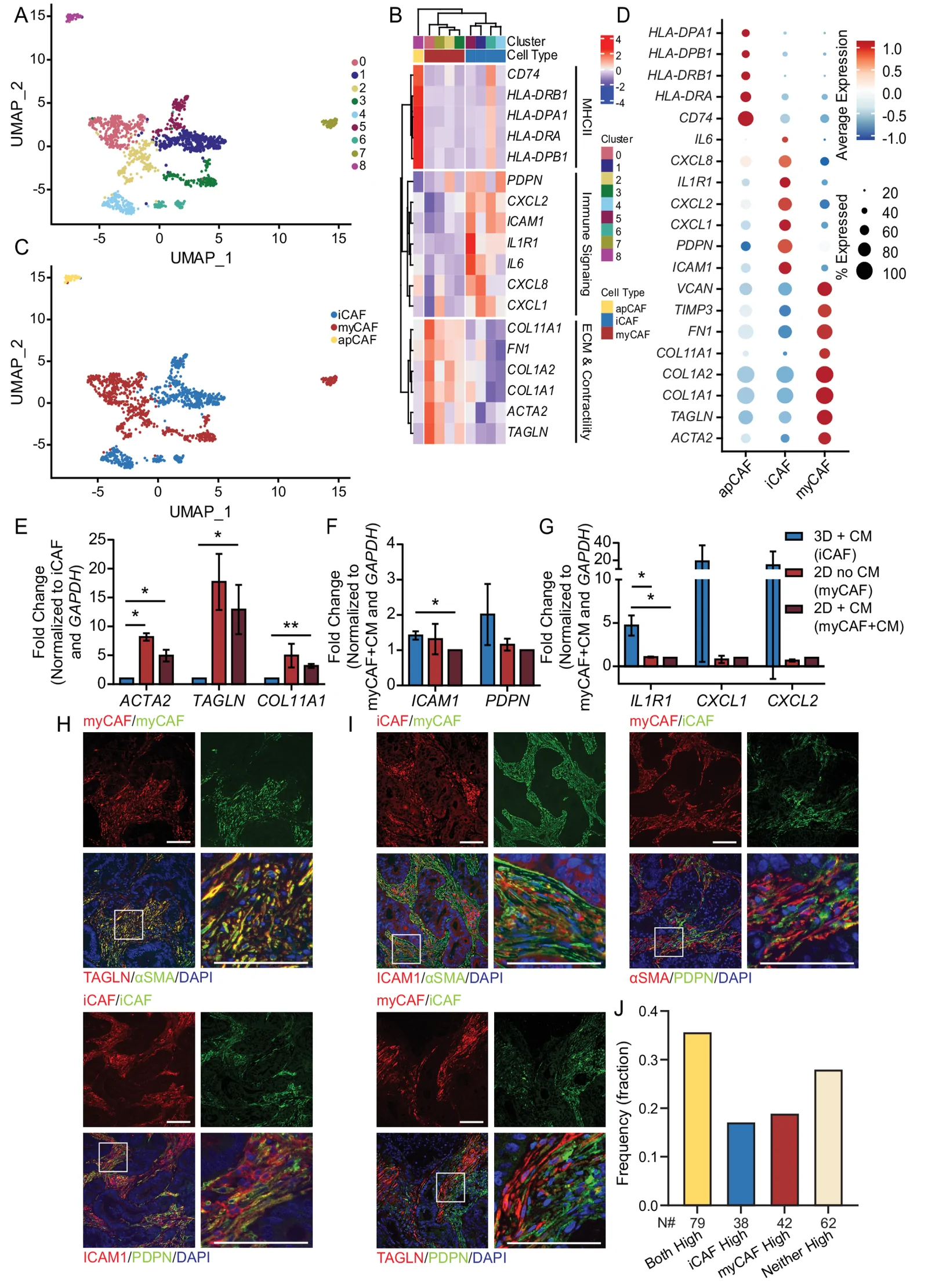
Novel markers for distinct CAF subtypes. **A** UMAP clustering of tumor fibroblasts in the SMC single-cell RNA sequencing cohort. **B** Heatmap of per-cluster average expression of genes related to previously defined CAF subtype functions, including expression of MHC II molecules, cytokine and chemokine secretion, and extracellular matrix remodeling. **C** Clusters relabeled as CAF subtypes. **D** Subtype-level expression of CAF markers. **E** and **F** Expression of (**E**) myCAF marker and functional, (**F**) iCAF marker, or (**G**) iCAF functional genes plated for 48 h in previously published culture conditions measured by RT-qPCR. **H** Dual immunofluorescence of myCAF markers αSMA and TAGLN and iCAF markers ICAM1 and PDPN. **I** Dual immunofluorescence of cross-subtype markers. Scale bars 100 μm. **J** Abundance of tumors high in CAF subtypes, determined by high staining of αSMA and TAGLN for myCAFs and ICAM1 and PDPN for iCAFs across 221 scoreable samples from 153 patients. *p < 0.05, **p < 0.01, ***p < 0.001, ns not significant

For in vitro assays, a minimum of three biological replicates were used for most experiments. The one exception is Fig. 1E-G, where the myCAF no CM condition had only two biological replicates due to low RNA yield, and repeat experiments could not be performed due to senescence of the primary line. For in vivo assays, sample sizes were chosen based on prior data of combination targeted therapies and anti-PD-1 studies to ensure 80% power at α = 0.05. Mice were randomized according to weight at study start, and final measurements of tumor size were blinded to treatment group.

### scRNA-seq re-analysis

The SMC raw reads and metadata [16] were downloaded from the NCBI Gene Expression Omnibus (GEO) database (accession GSE132465). Starting from data annotated as derived from cancer and of general cell type fibroblast, cells were normalized using Harmony [21] (version 1.0) and clustered and further analyzed using Seurat [22–25] (versions 2–4) in R [26] (version 4.1.0). Heatmaps were built using ComplexHeatmap [27, 28] (version 2.16.0).

### Human samples

All studies were completed following Institutional Review Board (IRB) approval with informed consent obtained from subjects. For immunofluorescence (IF) analysis of representative human tumors and derivation of CAF cell lines, human samples were acquired through the University of Wisconsin (UW) Translational Science BioCore (UW IRB#2016 − 0934), UW Molecular Tumor Board Registry (UW IRB#2014 − 1370), or UW Rectal and Anal Cancer Registry (UW IRB#2018 − 0149). For IF, tissues were fixed in 10% neutral-buffered formalin (Azer Scientific #NBF-4-G) overnight then stored in 70% ethanol until embedding in paraffin and sectioning. CAFs were derived from patient samples by digestion with collagenase and dispase, then cultured 2D with CAF feeding medium (1X DMEM (Corning #10-013-CV) supplemented with 20% FBS and antibiotics). Fibroblasts were confirmed by expression of *VIM* and lack of expression of *CDH1* by RT-qPCR.

For immunohistochemistry comparison across human tumors and quantification of immune cell infiltrates via IHC or IF, matched normal and CRC samples were obtained from 153 patients through protocol IRB#2016 − 0934. These patients distributed across stages of disease (I-IV), gender, race, and other clinical characteristics. Two representative cores of tumor tissue and one core of normal colon tissue were obtained for each patient in collaboration with surgical pathologists to ensure proper tissue sampling. These samples were distributed across two tissue microarrays.

### CAF polarization

CAFs were polarized as previously described [13, 18]. Briefly, CAFs were plated either 1:1 with Matrigel or in 2D, then fed either 1:1 CAF feeding medium: cancer feeding medium (Wnt feeding medium [29]) or 1:1 CAF feeding medium: cancer-conditioned medium. Cancer-conditioned medium was conditioned over 48 h from matched patient-derived cancer organoids, isolated and cultured as previously described [29]. Cells were collected 48 h after plating for analysis.

### RT-qPCR

Cells were trypsinized and collected in 1X PBS. RNA was isolated using the Qiagen RNEasy Mini Kit (Qiagen #74104) according to manufacturer instructions. 300-500ng of input RNA was used to make cDNA using Promega GoScript (Promega #A2791) according to manufacturer instructions. cDNA was diluted 1:5 for input into qPCR reactions. qPCR reactions were set up using the PR1MA No Rox qPCR MasterMix (MidSci #PR200-N-100) as follows: 10µL MasterMix, 2µL primers (final conc. 500 nM), 8µL diluted cDNA. Primers used are listed in table S2 in Additional File 1. qPCR was run on a BioRad CFX96 or BioRad CFXOpus 96 with the following program: 95 °C 3 min, 40 × (95 °C 10s, 58 °C 20s), followed by a melt curve. Values presented are 2^−ΔΔCq^ normalized to *GAPDH* or *Gapdh* expression and either control samples for treatment and PD-1 studies, or to CAF subtypes indicated in the figure for polarization studies. For myCAF and iCAF overall expression changes as presented in Fig. 5B, C_t_ values were averaged across all relevant markers for each tumor.

### Immunohistochemistry

Unstained tissue sections were deparaffinized and rehydrated according to standard procedures. Antigen retrieval was conducted by boiling for 30 min in citrate buffer (pH 6.0) for αSMA, TAGLN, PDPN, MYOCD, and MRTFA staining or EDTA buffer (pH 8.0) for CD8 and ICAM1. Primary antibodies and concentrations are listed in table S3 in Additional File 1. Sections were incubated in primary antibody diluted in PBS overnight before incubating with secondary antibody (MACH 2 Rabbit, RHRP520, BioCare Medical; SignalStain Boost IHC Detection HRP Rabbit, 8114, Cell Signaling Technologies, or MACH 2 Mouse, MHRP520, BioCare Medical) for 30 min at room temperature. Betazoid DAB buffer (BioCare Medical, SKU DS900) or SignalStain DAB (Cell Signaling Technologies #8059) were used to visualize staining, and hematoxylin (BioCare Medical SKU CATHE) was used for nucleic acid counterstain. CD4 and some CD8 staining was done at the UW Health Surgical Pathology laboratory on a BenchMark ULTRA automated system. Briefly, antigen retrieval is carried out for 64 min in buffer CC1 (Ventanta), and primary antibody (CD4 clone SP35, #790–4423, RRID: AB_2335982; CD8 clone SP57, #790–4460, RRID: AB_2335985, Ventana) was incubated for 32–20 min, respectively, and detected with Ventana UltraView Universal DAB Buffer (Ventana #760 − 500).

### Immunofluorescence

Unstained tissue sections were deparaffinized and rehydrated according to standard procedures. Antigen retrieval was conducted using buffers dependent upon primary antibodies listed in table S4 in Additional File 1. In the case of dual immunofluorescence where one antibody requires citrate buffer retrieval and another EDTA, EDTA was used for antigen retrieval. Autofluorescence was removed using TrueVIEW autofluorescence quenching kit (Vector Labs #SP-8400-15). Cells were permeabilized for transcription factors by adding 0.1% Triton X100 to primary antibody diluent. Primary antibodies were diluted in 5% BSA in either PBS or PBST and incubated overnight at 4 °C. Appropriate secondary antibodies labeled with Alexa Fluor 488 (Invitrogen, #A11001, RRID: AB_141367) and Alexa Fluor 647 (Invitrogen, A31573, RRID: AB_2536183) were incubated for 30 min at room temp. Nuclei were counterstained and mounted with VectaShield Vibrance Antifade Mounting Medium with DAPI (Vector Labs, #H-1800).

### Immunohistochemistry and immunofluorescence scoring

αSMA, TAGLN, ICAM1, and PDPN were scored on a 0–3 + scale, with a score of 0 being absent, 1 + being light and diffuse staining, 2 + being intermediate, and 3 + being dark, ubiquitous staining within the stromal compartment of tumor or normal samples. myCAF-high samples displayed an average score for αSMA and TAGLN of 2 or greater. Similarly, the average score for ICAM1 and PDPN was used to determine iCAF status. CD8 and CD4 stains were quantified as positive-staining lymphocytes in contact with tumor epithelium per high-powered (40X objective) field. MYOCD and MRTFA stains were quantified as positive-staining nuclei within the stromal compartment per high-powered field. Granzyme-B was scored as total number positive-staining foci appearing to emanate from a single cell, per high-powered field. Treg (CD4^+^FOXP3^+^) and Th1 (CD4^+^Tbet^+^) counts were scored as dual-positive cells per 20X objective field of view. All immunostaining was scored by at least two independent observers blinded to clinical information.

### TCGA

The Cancer Genome Atlas (TCGA) data was accessed via cBioPortal (cbioportal.org) [30]. PanCancer Atlas COADREAD data [31, 32] was separated into cohorts based upon mRNA RSEM V2 expression Z-scores. MyCAF high samples were those with a Z-score > 1 for both *ACTA2* and *TAGLN*, whereas iCAF high samples were those with a Z-score > 1 for both *PDPN* and *ICAM1.* Samples with a Z-score > 1 for either *PDPN* or *ICAM1* were excluded from the myCAF high group, and similarly samples with a Z-score > 1 for either *ACTA2* or *TAGLN* were excluded from the iCAF high group. Of 594 samples with RNA data in the COADREAD dataset, 24 were considered myCAF high by this analysis, and 9 iCAF high.

### CIBERSORTx

Normalized reads RSEM V2 for the myCAF high and iCAF high patients were accessed via cBioPortal. CIBERSORTx analysis was performed with the Impute Cell Fractions module. RSEM data for each of the 33 patients were used to create the mixture file [33]. The LM22 matrix [34] was used as the signature matrix file and the analysis was run in absolute mode. Data presented summarize the 24 myCAF high and 9 iCAF high patients’ individual results.

### Cell culture drug treatments

Nilotinib (HY-10159), dasatinib (HY-10181), and imatinib (HY-15463) were purchased from MedChemExpress. Drugs were stored no more than 6 months at − 80 °C and no more than 1 month at − 20 °C after reconstitution in DMSO. Drug treatment concentrations were based on C_max_ values for all drugs. Dasatinib treatments were done at 102 nM (100% C_max_) [35], 51 nM (50% C_max_) and 10.2 nM (10% C_max_); imatinib treatments at 5.26 µM [36], 2.63 µM, and 526 nM; and nilotinib treatments at 2.57 µM [37], 1.29 µM, and 257 nM. For all drug treatments presented, cells were plated and allowed to adhere and grow for 48–72 h. 10 mM stock solutions of drugs were diluted to the appropriate concentrations in fibroblast growth media then plated onto cells. Cells were re-treated with drugs after 48 h and collected 48 h later, for a total treatment time of 96 h. For RNA sequencing and western blot, only 100% C_max_ concentrations were used.

### Cell proliferation assays

For each replicate, cells were plated into two 96-well plates at 2000 cells/well. This number was empirically determined individually for each cell line via a dilution curve. On the first day of treatment, media was aspirated from one plate and replaced with media containing the appropriate concentration of dasatinib, imatinib, or nilotinib. This plate was treated as described above. Alongside the first treatment, the media in the second plate was replaced with media containing 10% WST-1 Cell Proliferation Agent (Sigma #11644807001). This plate was incubated at 37 °C and read on a Molecular Devices SpectraMax 384 Plus at 650 nm and 450 nm at designated time points up to 4 h. After 96 h, the treated plate was treated with WST-1 reagent and read similarly. The average readout for the day 0 reading at the same timepoint after WST-1 reagent was added in which the control on the day 4 timepoint reached an average absorbance of 0.8 was used to normalize cell proliferation.

### Western blot

Cells were collected after treatment by trypsinization followed by 1X PBS wash. Dry pellets were stored at − 80 °C until protein isolation. For protein isolation, cells were lysed in NP40 lysis buffer (Invitrogen #FNN0021) supplemented with protease inhibitor cocktail (Sigma #P8340) and PMSF (Sigma #P7626). 20 µg of lysate was denatured by boiling for 10 min in Laemelli sample buffer with BME. Proteins were separated on TGX AnykD gel (BioRad #4569036) in Tris/Glycine/SDS buffer, then transferred to an activated PVDF membrane in Tris/Glycine buffer with 20% methanol. Membranes were blocked in 5% skim milk in 1X TBST, then incubated overnight at 4 °C in primary antibody diluted in 5% BSA in TBST. Primary antibodies used were TIMP3 (Cell Signaling #5673, RRID: AB_10694530, 1:250), GAPDH (Cell Signaling, #8884, RRID: AB_11129865, 1:1000), and COL1A1 (Cell Signaling #72026, RRID: AB_2904565, 1:500). After overnight incubation, membranes were incubated at room temperature for 1 h in secondary antibody (Cell Signaling #7074; RRID: AB_2099233, 1:2000) in 5% BSA in TBST. Bands were visualized with SuperSignal West Dura Extended Duration Substrate (ThermoFisher #34076) on a BioRad ChemiDoc imaging system.

### Drug candidate identification

To identify drug candidates for altering the myCAF phenotype into the iCAF phenotype, the myCAF versus iCAF gene signature was generated using the FindMarkers command in Seurat. This gene signature was input into the Library of Integrated Network-Based Cellular Signatures (LINCS) L1000 Characteristic Direction Signature Search (L1000CDS^2^; https://maayanlab.cloud/L1000CDS^2^/) engine, set to find small molecules to reverse the gene signature.

### RNA sequencing

For RNA sequencing, three biological replicates for each treatment were collected and sequenced. Cells were collected after 96 h of treatment with 100% C_max_ dasatinib or nilotinib, or control medium. Cells were trypsinized, washed in PBS, then stored in RNAProtect Tissue Reagent (Qiagen #1018087) until RNA isolation. RNA isolation, quality control, library preparation, sequencing, and data analysis through differential expression analysis was performed by the UW Madison Gene Expression Center and Biotechnology Center Bioinformatics Core Facility (RRID: SCR_017799). Briefly, reads were trimmed using skewer [38] and mapped using STAR [39]. Reads were quantified using RSEM [40] and filtered and normalized in edgeR [41]. Differential expression analysis was also performed in edgeR.

### Gene set enrichment analysis

Gene set enrichment analysis (GSEA) was performed using GSEA software [42, 43]. Rank files were created using differential expression files from either RNA sequencing or accessed from cBioPortal for the TCGA datasets. Gene ranks were calculated as –log10(p-value) multiplied by the sign of the Log2 fold change. GSEA PreRanked was run against the Hallmark datasets [44].

### Mouse models and treatment

All animal studies were performed adhering to approved protocols by the Institutional Animal Care and Use Committee at the University of Wisconsin (Madison, WI) following the guidelines of the American Association for the Assessment and Accreditation of Laboratory Animal Care International. This study is reported in accordance with ARRIVE guidelines. In accordance with protocol endpoints, study mice were monitored daily and euthanized when visibly ill, including observations of failure to groom, reduced activity, anemia, body condition score less than 2, a 20% decrease in body weight, or other signs of discomfort or distress. *Fapb1:Cre*^*1*^*Apc*^*fl/+*^*Pik3ca*^*H1047R*^ (APPK) mice were bred [45] and genotyped as previously described [46–48]. Results presented are from three cohorts containing 18–21 mice each, totaling 58 mice. Nilotinib (MedChemExpress #HY-10159) was formulated in 0.5% hydroxypropylmethylcellulose (Sigma #09963 − 256) and 0.025% Tween80 (Sigma #P1754). Diluted nilotinib was sonicated to make a suspension and treated within an hour of formulation. Mouse anti-PD-1 (clone RMP1-14, BioXCell #BE0146, RRID: AB_10949053) and isotype rat IgG2a (clone 2A3, BioXCell #BE0089, RRID: AB_1107769) were purchased from BioXCell and diluted in the recommended PBS buffers (BioXCell #IP0070 and #IP0065, respectively). Nilotinib was dosed at 20 mg/kg daily by oral gavage and anti-PD-1 by intraperitoneal injection twice a week for 28 days. At the endpoint, the colon and the distal half of small intestine tissue were dissected, and tumors were counted and measured along their longest diameter via calipers.

### mPD-1 and anti-PD-L1 blocking antibody experiments

APPK-derived CAFs were isolated and cultured as described above for human primary derived CAFs. For mPD-1 experiments, cells were treated at listed concentrations of recombinant murine PD-1 (R&D Systems cat #9047-PD) for 48 h, then re-treated for another 48 h for a total of 96 h. For mPD-1/anti-PD-L1 experiments, cells were similarly treated with 4 µg/ml mPD-1 and either 10 µg/ml of a blocking anti-PD-L1 antibody (BioXCell #BE0383, RRID: AB_2927520) or rat IgG1 (BioXCell #BE0083, RRID: AB_1107784). Cells were collected and analyzed via qPCR as above.

### Statistical analysis

Statistical analyses were performed using GraphPad PRISM v. 10.0.2 for Windows (GraphPad Software, Boston, MA, www.graphpad.com) or R version 4.1.0 [26]. Wilcoxon Rank-Sum Tests were used to compare IHC and IF staining, all other statistical tests are Student’s T Tests.

## Results

### scRNA-seq analysis reveals molecularly identifiable CRC-specific markers of CAF subtypes

Recent studies across diseases highlight the importance of the diverse functions of CAFs in modulating the TME and response to therapies [12, 49]. A focused re-analysis of publicly available scRNA-seq data [16] was conducted to determine specific CAF subtypes and delineate their functional roles in the CRC TME. Fibroblasts from tumor samples separated into nine clusters (Fig. 1A). Markers from the differentially expressed gene list were used to annotate phenotypes of these clusters based on known CAF signatures (Fig. 1B). Four clusters were marked by high expression of *ACTA2*, *TAGLN* and extracellular matrix components such as collagens (Fig. 1B), which closely matches expression patterns indicative of myCAFs [12, 13, 15, 50]. The smallest cluster highlighted a rare population of fibroblasts expressing MHC II molecules (Fig. 1B), consistent with apCAFs [14]. The remaining four clusters highly expressed immune modulatory molecules, such as *CXCL1*,* CXCL2*, and *IL1R1* (Fig. 1B), indicative of iCAFs. The nine clusters were reclassified into 3 functional/phenotypic cohorts: myCAFs, iCAFs, and apCAFs (Fig. 1C) for further downstream and functional studies. MyCAF clusters expressed extracellular matrix molecules such as collagens and fibronectin, highlighting their function in ECM remodeling, while iCAF clusters expressed several chemokines and cytokines, highlighting their function in modulating immune cell signaling (Fig. 1D). To further investigate the roles of these CAF subtypes in CRC biology, markers delineating myCAF and iCAF phenotypes were chosen that could be used to identify these CAFs in vitro and *in vivo.* MyCAF clusters were marked by high expression of *ACTA2* (encoding α-smooth muscle actin) and *TAGLN*, whereas iCAFs were marked by high expression of *PDPN* and *ICAM1* (Fig. 1D). While the myCAF markers match validated markers in pancreatic cancer, these iCAF markers are distinct from those of pancreatic iCAFs [13, 15].

To test these markers in vitro, CAFs were polarized according to previously published techniques [13, 18] and evaluated for RNA expression of the above CAF subtype markers and associated functional genes. Plating CAFs as 2D cells increased expression of proposed myCAF markers *ACTA2* and *TAGLN*, as well as a functional marker *COL11A1*, matching the myCAF phenotype (Fig. 1E). Plating CAFs in a Matrigel matrix with cancer-conditioned media significantly increased expression of marker *ICAM1* and chemokine receptor *IL1R1*, with a trend towards increasing *PDPN* and chemokines *CXCL1 and CXCL2*, connecting the proposed iCAF markers to the iCAF phenotype (Figs. 1F, G). Six different human CRCs were stained via immunofluorescence to confirm that the proposed markers identify distinct populations of cells within the stromal compartment of CRC. CAF markers were largely expressed by cells located within the stromal compartment (Figs. 1H, I). Staining for myCAF markers αSMA and TAGLN significantly overlapped within CRC stroma, indicating co-expression in cells. Similarly, significant overlap was observed for iCAF markers PDPN and ICAM1 (Fig. 1H). Due to the plastic nature of CAF subtype expression [13], some cellular co-expression of myCAF markers with iCAF markers is expected. In these samples, there were few cells co-expressing αSMA and PDPN, αSMA and ICAM1, or TAGLN and PDPN, though cells expressing different phenotype markers were often adjacent and intermixed (Fig. 1I). These results were consistent across human CRCs (fig. S1 in Additional File 1). Taken together, these data identify the presence of both myCAFs and iCAFs within CRCs. These subtypes can be manipulated and are reliably marked in vitro and in human CRCs by *ACTA2/*αSMA and TAGLN, or PDPN and ICAM1, respectively.

To investigate variation in the abundance of CAF subtypes across CRCs and how the presence of different CAF subtypes may associate with other clinical features of CRC, we obtained 306 cancer samples across a cohort of 153 CRC patients. The abundance of CAF subtypes in CRC was evaluated using αSMA and TAGLN protein expression to mark myCAFs and PDPN and ICAM1 expression to mark iCAFs. For a detailed description of CAF scoring, see Methods. Of the 221 samples scoreable for all markers, 35.7% scored high for both iCAF and myCAF markers. Additionally, 19.0% of samples were high in myCAF markers and low in iCAF markers, and 17.2% were high in iCAF markers and low in myCAF markers (Fig. 1J). These results indicate that both subsets of CAFs studied are abundant within the TME of CRC, and that there is a lot of variation in the prevalence of these subtypes across patients. Demographic information across the different CAF subtypes can be found in table S1 (Additional File 1).

### CAF subtypes correlate with T cell infiltration into CRCs

**Fig. 2:**
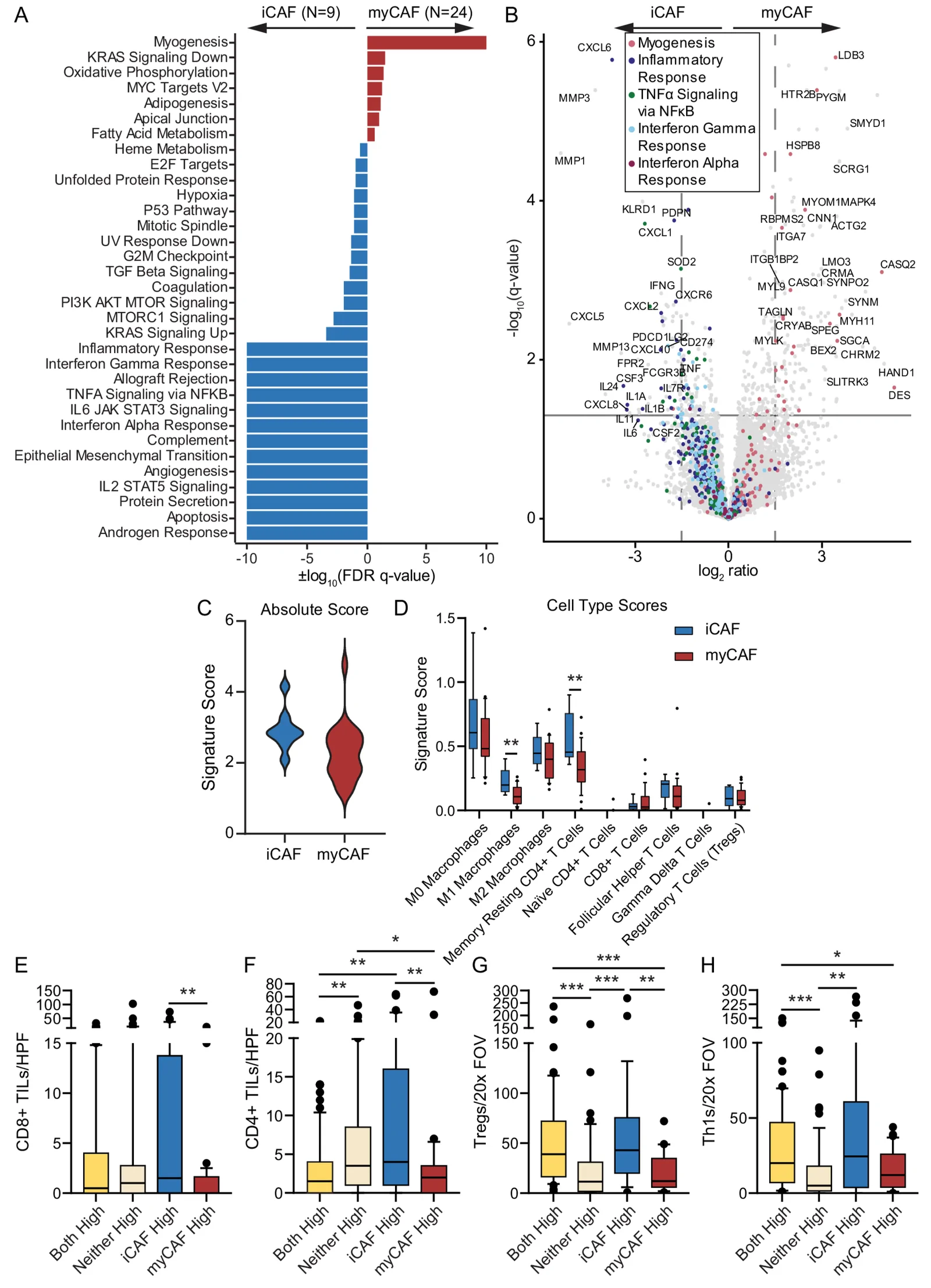
CAF subtypes are associated with differential immune infiltration in CRC. **A** Enrichment in Hallmark gene sets from differential expression results of iCAF-high and myCAF-high patients in the TCGA COADREAD dataset. **B** Expression of genes in the myogenesis, interferon alpha response, interferon gamma response, and inflammatory response pathways for myCAF-high vs. iCAF-high cancers. **C** Absolute score for immune infiltration across iCAF-high and myCAF-high patients in the TCGA COADREAD dataset in CibersortX analysis with the LM22 immune signatures. **D** Selected results for the LM22 immune cell subsets showing the differential abundance of macrophage and T cell subsets across iCAF-high and myCAF-high tumors. Infiltration of (**E**) CD8^+^ and (**F**) CD4^+^ lymphocytes across patient tumor samples. Lymphocytes were quantified as positive-staining lymphocytes in contact with tumor epithelium (tumor-infiltrating lymphocytes, TILs) per high-powered field (HPF) via immunohistochemistry. Infiltration of CD4^+^ T cell subsets (**G**) Tregs and (**H**) Th1 cells. Lymphocytes were quantified as total dual-positive staining cells expressing CD4 and (**G**) FOXP3 or (**H**) Tbet in a 20X objective field of view (FOV). *p < 0.05, **p < 0.01, ***p < 0.001

To further evaluate the different contributions of CAF subtypes to overall tumor biology, transcriptomic data available from The Cancer Genome Atlas (TCGA) PanCancer Atlas was analyzed to create a cohort of colorectal adenocarcinoma patients that were likely to be high either in only myCAFs or only iCAFs. Patients were stratified by either having high expression of both *ACTA2* and *TAGLN* for myCAF high tumors or *ICAM1* and *PDPN* for iCAF high tumors (see Methods for details). Importantly, samples with high expression of *ICAM1* or *PDPN* were removed from the cohort of myCAF high tumors and samples with high expression of *ACTA2* and *TAGLN* were removed from the cohort of iCAF high tumors to best separate the roles of these different CAF subsets. Gene Set Enrichment Analysis of the differential expression results from these two separate tumor sets showed high upregulation of immune-related pathways such as interferon alpha response, interferon gamma response, and inflammatory response in iCAF high tumors, whereas myCAF high tumors had high expression of myogenesis-related genes (Fig. 2A). Specifically, myCAF high tumors had high expression of integrin-related genes *ITGA7* and *ITGB1BP2*, essential for cell-ECM crosstalk [51], and *CRYAB*, previously associated with epithelial-mesenchymal transition in CRC [17]. iCAF high tumors had high expression of chemokines, such as *CXCL1*,* CXCL2*,* CXCL8*, and *IFNG* (Fig. 2B).

The function of CAFs in modifying the immune system is of great interest due to its relevance to immunotherapies. Subtypes of CAFs contribute to the infiltration and polarization of different immune cells through several mechanisms [12]. To further characterize the immune microenvironment associated with different CAF subtypes, the abundance of immune cells within the tumors of the myCAF high versus (vs.) iCAF high TCGA cohorts was estimated with CibersortX (Figs. 2C-D). The iCAF high cohort had a higher overall abundance of immune cells, with an average CibersortX absolute score of 2.9 for iCAF high tumors vs. 2.3 for myCAF high tumors (*p* = 0.009; Fig. 2C). Within subsets of immune cells, differences in the infiltration of some T cell and macrophage subtypes were observed (Fig. 2D). In particular, iCAF high tumors displayed a statistically significant higher abundance of anti-tumor M1-like macrophages and memory resting CD4^+^ T cells (Fig. 2D).

While there was no significant difference in CD8^+^ T cells in this analysis, the activity of these cells is highly dependent on proximity to cancer cells, which cannot be measured in RNA-based studies. To understand the spatial context of T cell dynamics in tumors predominant in different CAF subtypes, T cell infiltration was evaluated in the cohort of CRC patients using immunohistochemistry and immunofluorescence. Samples that were high in iCAFs tended to have the highest infiltration of CD8^+^ and CD4^+^ T cells, with the iCAF high only subset consistently having the highest infiltration of T cells (Figs. 2E-H). MyCAF high only tumors had the least abundance of CD8^+^ T cells infiltrating the epithelial compartment (tumor infiltrating lymphocytes [TILs]; Fig. 2E), whereas the iCAF high only tumors had the greatest quantity of CD8^+^ TILs. iCAF high tumors displayed significantly higher infiltration of CD4^+^ T cells (Fig. 2F), including both Tregs (Fig. 2G) and Th1 cells (Fig. 2H). With these data we hypothesized that altering the CAFs displaying a myCAF phenotype into a more iCAF-like phenotype may improve anti-tumor immune response by increasing infiltration of cytotoxic CD8^+^ T cells, and this resulting infiltration may improve response to T cell-based immunotherapies, such as anti-PD-1 agents.

### The tyrosine kinase inhibitor nilotinib decreases expression of myCAF genes and functional markers

**Fig. 3:**
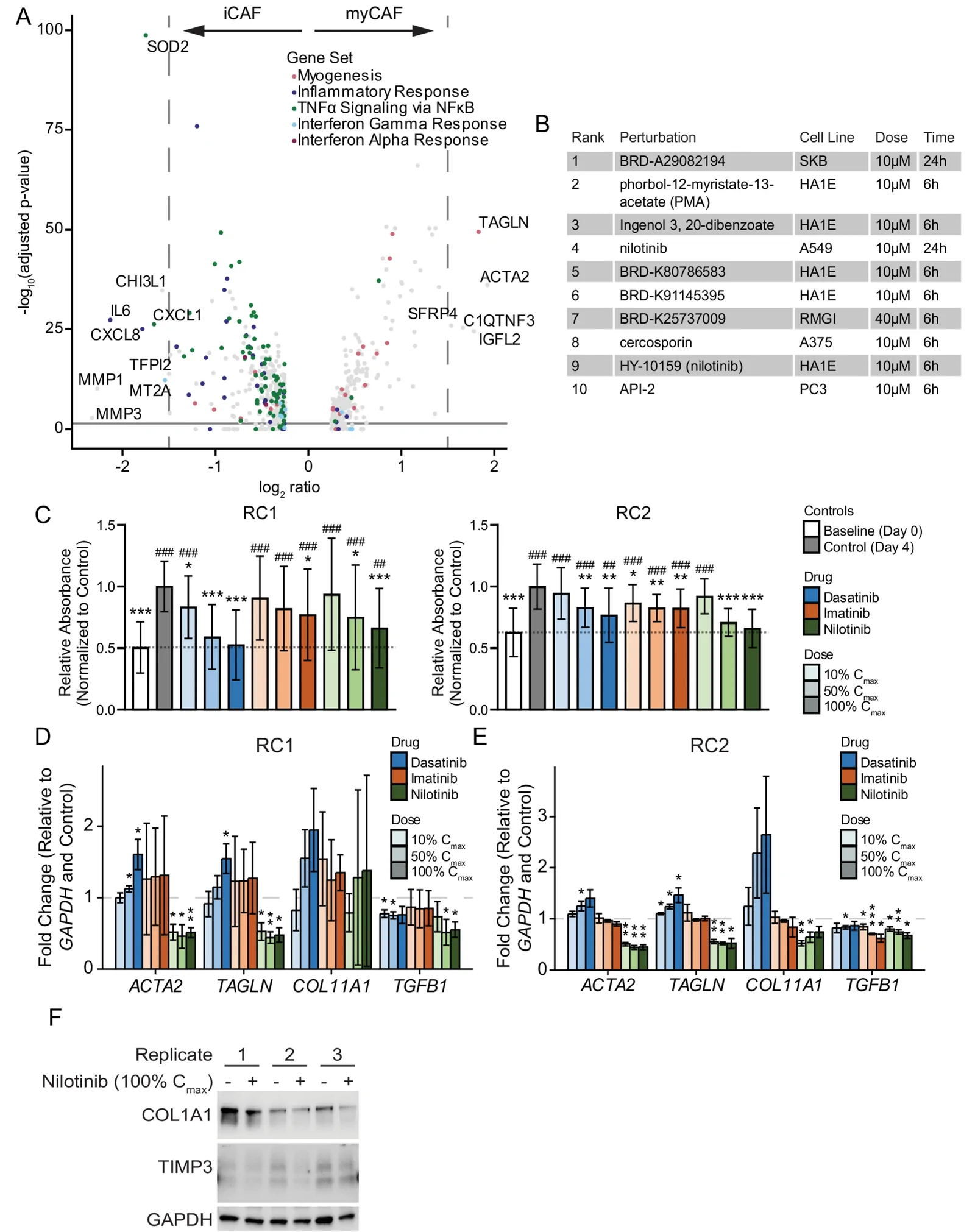
Nilotinib decreases the expression of myCAF genes and extracellular matrix remodeling proteins in patient-derived CAFs. A Differentially expressed genes between iCAF and myCAF cells in scRNA-seq. B Top ten drugs predicted to reverse the differential myCAF: iCAF gene signature via L1000CDS^2^. C Two patient-derived rectal cancer CAF lines were treated with 10%, 50%, and 100% physiological Cmax of nilotinib and related tyrosine kinase inhibitors imatinib and dasatinib for 96 h, replacing treatment at 48 h, and measured for change in cell viability using WST-1 proliferation reagent compared to cells at time of first treatment. *p < 0.05, **p < 0.01, ***p < 0.001 relative to control (untreated cells) at 96 h, #p < 0.05, ##p < 0.01, ###p < 0.001 relative to baseline control at 0 h. D-E Average change in expression of myCAF genes ACTA2, COL11A1, and TAGLN and myCAF-associated growth factor TGFB1 over 96 h in (D) RC1 and (E) RC2 cells measured by RT-qPCR. *p < 0.05, **p < 0.01, ***p < 0.001 relative to control (untreated cells) at 96 h. F Western blot of myCAF functional proteins COL1A1 and TIMP3 in RC2 cells across three biological replicates treated with 100% Cmax nilotinib for 96 h

To better evaluate the cell-intrinsic functions of CAFs that may alter the immune infiltration of CRCs, differential expression analysis was performed on the scRNA-seq dataset between myCAFs and iCAFs (Additional File 2). Overlap with the most differentially expressed genes revealed that myCAFs were strongly associated with epithelial to mesenchymal transition and extracellular matrix gene sets, whereas iCAFs were again strongly associated with expression of inflammatory pathways (Fig. 3A and fig. S2 in Additional File 1). Given these data and the relationships of myCAFs and iCAFs with CD8^+^ lymphocyte infiltration (Fig. 2), we next aimed to identify potential therapeutics that could decrease the myCAF phenotype while increasing the iCAF phenotype. It was important to focus on therapeutics that could alter the phenotype and tumor-promoting functions of CAFs as opposed to eliminating them, as therapies that have focused on eliminating fibroblasts in the TME in the past have had less than favorable results [52, 53]. The L1000CDS^2^ tool from the LINCS project predicted nilotinib, a tyrosine kinase inhibitor, as a top candidate to reverse the myCAF phenotype (Fig. 3B).

Nilotinib is FDA-approved as a BCR-ABL inhibitor but is functionally a tyrosine kinase inhibitor that may have many potentially favorable off-target effects. Nilotinib and two other clinical BCR-ABL tyrosine kinase inhibitors, imatinib and dasatinib, were investigated. Two different primary human CRC-derived CAF cultures were plated and treated under myCAF-promoting adherent conditions with physiologically relevant concentrations of these inhibitors over 96 h. High concentrations of inhibitors led to a modest decrease in viability compared to untreated cells after 96 h, but still greater viability of the cultures than baseline (Fig. 3C). Imatinib had no effect on the RNA expression of myCAF markers, whereas dasatinib treatment increased the expression in a dose-dependent manner (Figs. 3D-E; *ACTA2* fold change [2^−ΔΔCq^] 1.00 [*p* = 0.96], 1.12 [*p* = 0.04], and 1.60 [*p* = 0.04] for RC1, 1.10 [*p* = 0.08], 1.25 [*p* = 0.04], 1.40 [*p* = 0.05] for RC2, and *TAGLN* fold change 0.91 [*p* = 0.5], 1.15 [*p* = 0.3], 1.55 [*p* = 0.04] for RC1 and 1.10 [*p* = 0.01], 1.24 [*p* = 0.01], and 1.46 [*p* = 0.03] for RC2 for 10%, 50%, and 100% C_max_, respectively). Nilotinib successfully reduced the expression of myCAF markers *ACTA2* and *TAGLN* by half across all concentrations (Figs. 3D-E; *ACTA2* fold change 0.52 [*p* = 0.02], 0.46 [*p* = 0.03], and 0.51 [*p* = 0.007] for RC1, 0.51 [*p* = 0.001], 0.44 [*p* = 0.002], 0.45 [*p* = 0.004] for RC2, and *TAGLN* fold change 0.53 [*p* = 0.02], 0.44 [*p* = 0.007], 0.48 [*p* = 0.01] for RC1 and 0.56 [*p* = 0.002], 0.52 [*p* = 0.001], and 0.52 [*p* = 0.01] for RC2 for 10%, 50%, and 100% C_max_, respectively). Treatment with all three inhibitors reduced the expression of iCAF genes (fig. S3 in Additional File 1). Additionally, treatment with nilotinib successfully reduced protein expression of extracellular matrix molecules (COL1A1) and remodeling proteins (TIMP3) functionally associated with myCAFs (Fig. 3F). While treatment with nilotinib failed to induce the iCAF phenotype, the myCAF phenotype is still associated with reduced immune infiltration (Fig. 2E), and we decided to move forward with this treatment due to the suppression of the myCAF phenotype alone.

### MYOCD and MRTFA are potential downstream targets of nilotinib that may drive the myCAF phenotype

To evaluate possible mechanisms by which nilotinib decreases the myCAF phenotype, RNA-seq was performed on two primary patient-derived CAF lines from rectal cancer patients. Since dasatinib has a large overlap in kinase targets with nilotinib but induced opposite effects on the myCAF phenotype, dasatinib treatment was included to further narrow the potential myCAF-specific changes in nilotinib action. Cells were treated for 96 h with dasatinib, nilotinib, or control media. Each cell line exhibited a unique transcriptome (fig. S4A in Additional File 1). Additionally, RC1, which had higher baseline expression of *ACTA2* and *TAGLN*, more strongly downregulated myCAF genes upon nilotinib treatment, whereas RC2 more strongly upregulated myCAF genes upon dasatinib treatment (figs. S4B-C in Additional File 1).

**Fig. 4:**
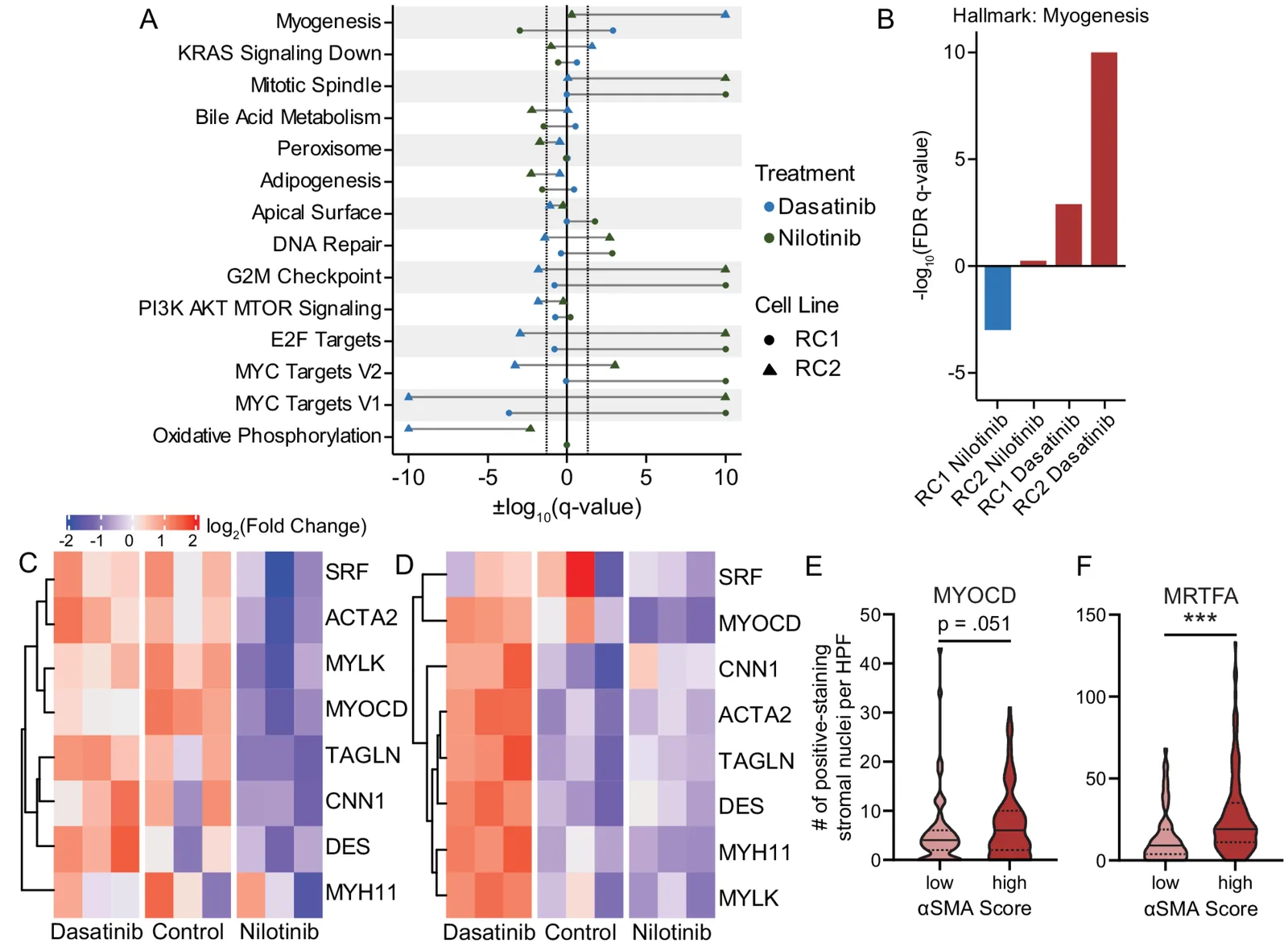
MYOCD and MRTFA are potential drivers of the myCAF phenotype. **A** Gene set enrichment analysis of hallmark gene sets on RC1 and RC2 treated with either dasatinib or nilotinib. Gene set enrichment was performed on the differential expression of each treatment versus control. Values graphed are the –log10(q-value) for each upregulated gene set, and the log10(q-value) of each downregulated gene set. Dotted lines represent q = 0.05. **B** Myogenesis gene set enrichment for each treatment. **C**-**D** RNA expression of *MYOCD*,* MRTFA*, and transcription cofactor *SRF*, as well as MYOCD and MRTFA target genes *ACTA2*,* TAGLN*,* CNN1*,* DES*, and *MYH11* in (**C**) RC1 and (**D**) RC2 cells treated with dasatinib, nilotinib, or untreated. **E**-**F** Quantification of (**E**) MYOCD and (**F**) MRTFA-positive staining stromal nuclei across human tumors also staining low and high for αSMA. Nuclei are scored as number of positive-staining nuclei per 40x high-powered field. *p < 0.05, **p < 0.01, ***p < 0.001

To narrow possible mechanisms of nilotinib action on the myCAF phenotype, differential expression of nilotinib- and dasatinib-treated cells were analyzed for gene set enrichment (Figs. 4A-B and figs. S4D-G in Additional File 1). Because dasatinib and nilotinib have opposite effects on the myCAF phenotype, we focused on gene sets in which these two drugs had opposite effects (Fig. 4A). Dasatinib treatment significantly increased myogenesis related genes in both cell lines, whereas nilotinib treatment decreased this pathway (Fig. 4B). Major drivers of myogenesis include serum response factor co-factors MYOCD and myocardin related transcription factors A and B (MRTFA/B) [54, 55], and both MYOCD and MRTFA are known to be major positive regulators of *ACTA2* and *TAGLN* expression [56]. *MYOCD* and several MYOCD/MRTFA response genes were significantly downregulated upon nilotinib treatment (Figs. 4C-D), indicating that one or both transcription factors may be key to nilotinib’s ability to regulate the myCAF phenotype.

To evaluate the relationship between MYOCD or MRTFA activation and the myCAF phenotype, the abundance of myCAF markers was compared with MYOCD and MRTFA in the UW cohort. Since there are no antibodies against phosphorylated MYOCD and MRTFA readily available, activation of MYOCD and MRTFA was estimated by quantifying positive-staining nuclei within the stromal compartment of tumors, taking advantage of the nuclear translocation upon phosphorylation observed for both transcription factors [57, 58]. There was a correlation between transcription factor activation and αSMA abundance in cancers, as high amounts of αSMA staining correlated with a higher median number of MYOCD- (Fig. 4E) and MRTFA-positive stromal nuclei per high powered field (Fig. 4F), strengthening the relationship between MYOCD/MRTFA and the myCAF phenotype.

### Nilotinib treatment significantly alters the myCAF phenotype in vivo

**Fig. 5:**
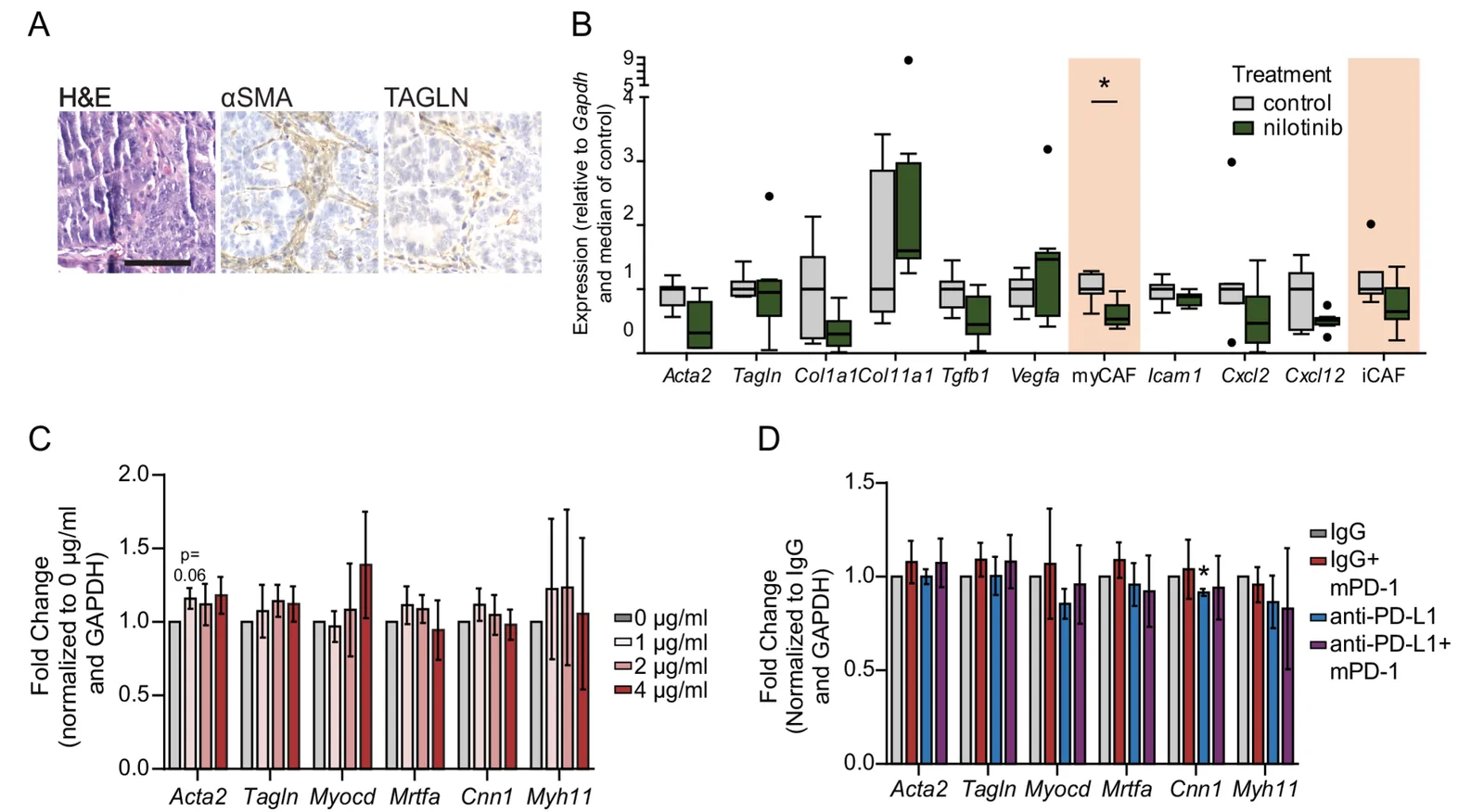
Single agent nilotinib decreases the myCAF phenotype in vivo. **A** H&E, αSMA, and TAGLN staining of *Fc*^*1*^*Apc*^*fl/+*^*Pik3ca*^*H1047R*^ (APPK) mice. Scale bar 100 μm. **B** Expression of key myCAF and iCAF genes in 12 APPK tumors treated with vehicle control (*n* = 5 tumors from 3 mice) or 20 mg/kg nilotinib (*n* = 7 tumors from 3 mice) daily for 10 days. **C**-**D** RNA quantification of *Myocd*,* Mrtfa*, MYOCD/MRTFA target genes *Myh11* and *Cnn1*, and myCAF genes *Acta2* and *Tagln* in APPK-derived CAFs after treatment with (**C**) 0–4 µg/ml mPD-1 or (**D**) 10 *p < 0.05 relative to IgG

Next it was determined whether nilotinib could alter CAF phenotypes *in vivo.* To identify an immunocompetent mouse model with high myCAF infiltration at baseline, a panel of syngeneic flank injection models and genetically engineered mouse models was surveyed for expression of αSMA and TAGLN (Fig. 5A and fig. S5 in Additional File 1). Syngeneic injection models showed little to no staining of myCAF markers (fig. S5). Genetically engineered mouse models harboring mutations in either *Apc* and *Kras* or *Apc* and *Pik3ca* showed higher staining of myCAF markers (Fig. 5A and fig. S5), as well as flank injections of organoids derived from *Apc* and *Pik3ca*-mutant cancers injected with or without additional fibroblasts (fig. S5). *Fc*^*1*^*Apc*^*fl/+*^*Pik3ca*^*H1047R*^ (APPK) mice [45, 59] were chosen for these studies (Fig. 5A).

To test the effect of nilotinib in vivo, APPK mice were allowed to age to develop invasive cancers and then treated with nilotinib or vehicle control for 10 days. Mice treated with nilotinib showed decreased expression of myCAF marker and functional genes (*p* = 0.04) and a trend towards decreased expression in iCAF marker and functional genes (Fig. 5B). Specifically, expression of *Acta2* and *Col1a1* was reduced by about 70% (2^−ΔΔCt^ 0.30 and 0.29 relative to median of control tumors), *Col1a1*, and *Tgfb1* by 56% (0.44) in nilotinib treated tumors, highlighting the in vivo effect of nilotinib on altering CAF phenotypes.

Because the myCAF phenotype correlates with decreased CD8^+^ lymphocyte infiltration, and nilotinib treatment decreases the function of myCAFs in vivo, we hypothesized that nilotinib treatment would lead to improved treatment response when combined with immunotherapy by increasing CD8^+^ lymphocyte infiltration. Before evaluating the combination therapy, the potential effect of anti-PD-1 alone on CAFs was investigated, as recent literature indicates this therapy may have a favorable effect on CAF phenotypes directly. Some studies have suggested that the TGF-β pathway can be activated by PD-L1 [60], and that treatment with anti-PD-L1 therapies can decrease myofibroblast differentiation in models of lung fibrosis [61]. To investigate whether PD-1 had the potential to drive the myCAF phenotype by activation of MYOCD/MRTFA, APPK-derived CAFs were treated with murine PD-1 (mPD-1) and measured its effects on myCAF and MYOCD/MRTFA target genes. Addition of mPD-1 to APPK CAFs trended towards an increased expression of *Myocd*,* Acta2* and *Tagln* in a dose-dependent manner, though not *Mrtfa*,* Myh11*, or *Cnn1* (Fig. 5C). To further evaluate the potential role of PD-1 in activating the myCAF phenotype, APPK-derived CAFs were treated with mPD-1 in the presence of anti-PD-L1 blocking antibody (Fig. 5D). Again, addition of mPD-1 without anti-PD-L1 showed a trend towards increasing expression of myCAF genes and *Myocd*. Interestingly, for *Myocd* and target gene *Myh11*, there was a trend towards decreasing expression with anti-PD-L1, even in the absence of mPD-1, and a significant decrease in expression of *Myocd* target *Cnn1.*

### Nilotinib treatment modestly but significantly improves response to anti-PD-1 in vivo

**Fig. 6:**
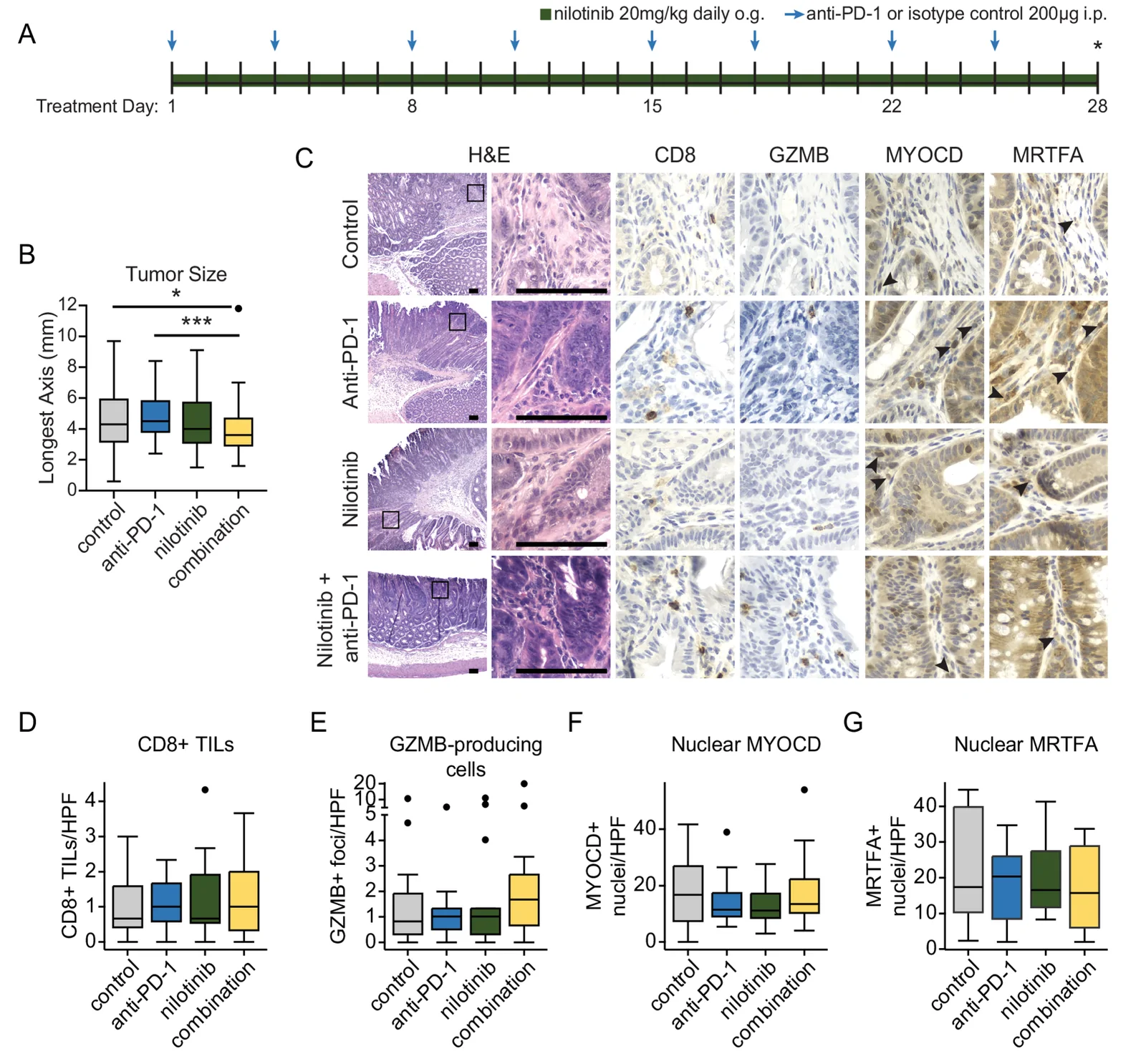
Nilotinib and anti-PD-1 combination increases granzyme B and decreases stromal nuclear myocardin. **A** Treatment schema for nilotinib and anti-PD-1 combination. Mice were treated daily with 20 mg/kg nilotinib or vehicle, and twice weekly with anti-PD-1 or isotype control for 28 days. **B** Longest diameter of tumors in the S3 and S4 segment of the small intestine and throughout the colon after treatment. **C** Representative low-zoom H&E, high-zoom H&E, and IHC staining for CD8, GZMB, MYOCD, and MRTFA in control, anti-PD-1 treated, nilotinib treated, and combination treated mice. Arrows indicate positive-staining stromal nuclei. **D** CD8 staining was quantified as positive staining lymphocytes in contact with cancer epithelium (tumor-infiltrating lymphocytes, TILs). **E** Granzyme B staining was quantified as positive staining foci appearing to emanate from a single cell. **F** MYOCD and (**G**) MRTFA were quantified as positive-staining stromal nuclei. All stains were quantified as an average of 3 high-powered fields per tumor. *p < 0.05, ***p < 0.001. All scale bars 100 μm

A 28-day study in APPK mice combining nilotinib and anti-PD-1 therapy was conducted to evaluate the impact of nilotinib treatment on immune infiltration and immunotherapy response (Fig. 6A). Therapy was well-tolerated and mouse weights remained consistent throughout the study (fig. S6 in Additional File 1). Combination treatment significantly reduced tumor size at the end of the 28-day study (*p* < 0.05; Fig. 6B). Overall, combination treated tumors had a flatter morphology (Fig. 6C). To determine whether nilotinib treatment altered the immune infiltration and activation of CD8^+^ T cells, the number of CD8^+^ tumor infiltrating lymphocytes and granzyme B-producing cells per high-powered field were quantified in treated and control mice (Figs. 6C-E). Though not statistically significant, trend was observed with combination nilotinib and anti-PD-1 treatment increasing the infiltration of CD8^+^ lymphocytes (Fig. 6D; median 0.7 CD8^+^ TILs/HPF in control tumors vs. 1.0 CD8^+^ TILs/HPF in combination, p = n.s.). A trend toward treatment increasing the expression of granzyme B was also observed (Fig. 6E; median 0.8 granzyme B-producing cells/HPF in control tumors vs. 1.7 granzyme B-producing cells/HPF in combination, p = n.s.).

To evaluate whether nilotinib treatment can decrease activation of MYOCD or MRTFA in vivo, nuclear staining of MYOCD and MRTFA was quantified in tumor tissues (Figs. 6C, F-G). Nilotinib treatment alone decreased the number of MYOCD-positive stromal nuclei (median 16.7 MYOCD+ nuclei/HPF in control vs. 11.2 for nilotinib treatment), though this finding was not statistically significant. Interestingly, there was a similar decrease in MYOCD-positive nuclei in the anti-PD-1 treated tumors (11.5 MYOCD+ nuclei/HPF; Fig. 6F). There was no trend in the number of MRTFA-positive stromal nuclei (Fig. 6G).

## Discussion

CAFs make up the bulk of the tumor stroma and play an important role in the cellular signaling that across the microenvironment of the tumor [11, 12]. A deeper understanding of the CAF composition in cancers, including their prevalence, functions, and the mechanisms behind these functions will improve our ability to capitalize on their presence in the TME. Several studies have evaluated various relationships between specific CAF-expressed factors and CRC biology [18, 19], but an understanding of how each of these separate factors are controlled and how they interact together to influence tumor biology is necessary to fully understand how CAFs may be altered for patient benefit.

Here we performed a focused re-analysis of scRNA-seq of tumor fibroblasts from CRC patients, identifying functional groups of CAFs similar to myCAFs, iCAFs, and apCAFs described in different diseases [11, 13–15, 20]. Markers of the myCAF and apCAF functional clusters were consistent with previous findings. While the remaining CAF clusters displayed high expression of chemokines and cytokines identifying them as iCAFs, these cells were best marked by *PDPN* and *ICAM1.* While *PDPN* has previously been described as either a pan-CAF or subtype marker [12, 13, 15], this is the first time to our knowledge that *ICAM1* has been identified as a subtype marker. It is not surprising to find a novel marker, as previous work has not focused on CRC-specific markers, and research in other diseases often finds disease-specific markers of CAFs [20]. These findings highlight that the functions of CAFs in CRC are largely similar to those found in other disease types, but is important to identify disease-specific markers of CAFs.

Importantly, these novel markers were used to correlate high stromal expression of CAF markers to clinically relevant markers of disease subtypes. myCAF-high tumor samples were largely immune-excluded, whereas iCAF-high tumors were immune infiltrated, providing a clinically actionable target to improve therapy. These data led to the identification of nilotinib as a potential therapeutic agent to favorably alter CAF phenotypes. Nilotinib and the related tyrosine kinase inhibitors imatinib and dasatinib are FDA approved for clinical use in acute myeloid leukemia for inhibition of the BCR-ABL fusion gene. However, these drugs have other targets and are used to treat gastrointestinal stromal tumors [62], showing potential for altering mesenchymal phenotypes within cancers. Dasatinib has been investigated in combination with anti-PD-1, though the models used in these studies often have limited stroma and do not consider the potential contribution of CAFs [63, 64]. While CAFs have roles that protect the tumor, they also restrict tumor growth and regulate differentiation, making depletion an ineffective treatment strategy [52, 53]. Importantly, these therapies did not have a significant impact on CAF viability. Both in vitro and in vivo, nilotinib treatment decreased the expression of myCAF markers *ACTA2* and *TAGLN* and decreased expression of extracellular matrix molecules and remodelers, indicating its potential use as a CAF-remodeling agent. When combined with anti-PD-1 therapy in vivo, nilotinib treatment not only led to reduced tumor size but tended to increase infiltration of CD8^+^ T cells and granzyme B-producing cells. Interestingly, we measured response to nilotinib in two different primary-derived rectal cancer lines, and found varying degrees of response to treatment, which may have been related to a differential tendency towards myCAF phenotype at baseline. Further experimentation in more fibroblast lines is necessary to determine whether there is a specific subset of disease for which this treatment strategy would be most effective.

Finally, these investigations pinpointed MYOCD and MRTFA as potential drivers of the myCAF phenotype whose activation is altered by nilotinib treatment. Nilotinib treatment decreased expression of the myogenesis gene set, a major downstream pathway for these transcription factors [54, 55], and the RNA expression of *MYOCD* and MYOCD/MRTFA target genes. Increased nuclear expression of both MRTFA and MYOCD was associated with high expression of the myCAF marker αSMA in human tissues, and treatment with nilotinib or anti-PD-1 therapy led to a decrease in nuclear expression of MYOCD in APPK tumors, though this finding was not statistically significant. The PD-1/PD-L1 axis may have a direct effect on myCAF phenotype, as PD-L1 can induce a myofibroblastic phenotype through stabilization of TGF-β [60], and myofibroblast differentiation can be attenuated with PD-L1 antibody therapy [61]. When treated with mPD-1, APPK-derived CAFs showed a trend towards decreased expression of *Myocd* as well as myCAF markers *Acta2* and *Tagln*, indicating PD-1 could be influencing the myCAF phenotype through activation of MYOCD. Overall, these data point towards a mechanism by which nilotinib treatment decreases the extracellular matrix remodeling functions of myCAFs by decreasing activation of MYOCD and/or MRTFA. Since the findings herein were not statistically significant and largely correlative, further mechanistic investigation is needed to determine whether this is a true biological phenomenon.

In this study, we identified three major subtypes of CAFs seen previously. However, because apCAFs are a rare population that would be difficult to identify, we did not consider this CAF population and its effects in downstream applications. While this manuscript focuses on the effects of nilotinib treatment on myCAFs due to the known links between CD8^+^ T cell infiltration and immunotherapy response [65, 66], the role of iCAFs and other immune cells may have significant impact on response. While the presence of myCAFs is associated with immune exclusion, iCAF-associated genes are correlated with high infiltration of tumor-promoting immune cells [12, 67]. While the iCAF phenotype was associated with increased infiltration of CD8^+^ T cells and Th1 helper T cells, it was also associated with higher infiltration of Tregs, highlighting the complex role of iCAFs in altering the immune microenvironment of tumors. Despite the initial hypothesis that nilotinib treatment would cause a phenotypic switch from the myCAF phenotype to an iCAF phenotype, nilotinib treatment instead decreased expression of both myCAF and iCAF genes. In this manuscript, we decided to focus examining the effects of decreasing myCAF phenotype on immune response in light of these results. The question of whether a full phenotypic switch to an iCAF state would further improve immune infiltration in a net-positive manner given the complex relationship to competing immune subsets remains as an intriguing future direction. Further, RNA sequencing results indicated that nilotinib treatment decreased expression of several immune-related pathways. Further experimentation is necessary to determine whether these results indicate an overall pro-tumor or anti-tumor change in TME. These complex immunological interactions may explain why changes in CD8^+^ T cell infiltration upon nilotinib treatment were not as significant as expected, warranting further investigation into changes in treated tumors, including how nilotinib treatment interacts with other CAF subtypes and immune cells. In these studies, addition of nilotinib statistically significantly improved tumor response to anti-PD-1 alone, though this effect was modest. Studies into the role of other immune cells in tumor response, as well as whether other inhibitors that may be able to more significantly alter CAF phenotypes would more greatly improve response are of great interest. While not tested here, there are currently MRTFA inhibitors available [68, 69] which may more specifically inhibit the immune-exclusionary functions of myCAFs.

Overall, these studies show how targeting the functions of CAF subtypes rather than single markers can alter TMEs and improve treatment response. These studies present novel markers of CRC CAF subtypes with similar functions to previous literature in other diseases and go beyond previous work by identifying and manipulating these CAFs in biological samples. Further, we identified nilotinib as a CAF-altering agent that, without killing CAFs and eliminating their tumor-restrictive functions, alters CAF phenotypes to improve responses to therapies. We identified a decrease in activation of MYOCD and MRTFA downstream of nilotinib treatment that is related to functions of the myCAF phenotype. Further investigation into the kinase targets of nilotinib upstream of MYOCD and MRTFA may help pinpoint more specific targets and improve response. Finally, investigation into the changes conferred by nilotinib on the overall immune microenvironment and iCAF function is of great interest and may highlight additional ways in which nilotinib treatment improves anti-tumor immune response.

## Conclusions

Utilizing inhibitors to regulate cancer-associated fibroblast phenotypes can alter the tumor microenvironment in a favorable way to improve response to anti-cancer therapies, such as anti-PD-1. The immune-exclusionary myCAF phenotype may be driven by MYOCD and MRTFA, and further research should focus on this mechanism to develop CAF-targeting therapeutic strategies.

## Supplementary Information

Additional file 1. Supplementary figures and tables.

Additional file 2. Differentially expressed myCAF and iCAF Genes.

## Data Availability

The single cell dataset analyzed during the current study is available in the Gene Expression Omnibus (GEO) accession #GSE132465. TCGA datasets are available at cbioportal.org. RNA sequencing datasets generated in this study are available in the GEO database, accession #GSE316373.
