# Supplementary material for: Suppression of the myofibroblastic cancer-associated fibroblast phenotype to enhance anti-PD-1 response
Source: J Exp Clin Cancer Res. 2026 Jul 10;45:208. doi: 10.1186/s13046-026-03779-3 (PMC13640252; doi:10.1186/s13046-026-03779-3)
Supplement: Supplementary file 1 [file 13046_2026_3779_MOESM1_ESM.pdf]

Supplemental Figures and Tables

Fig. S1

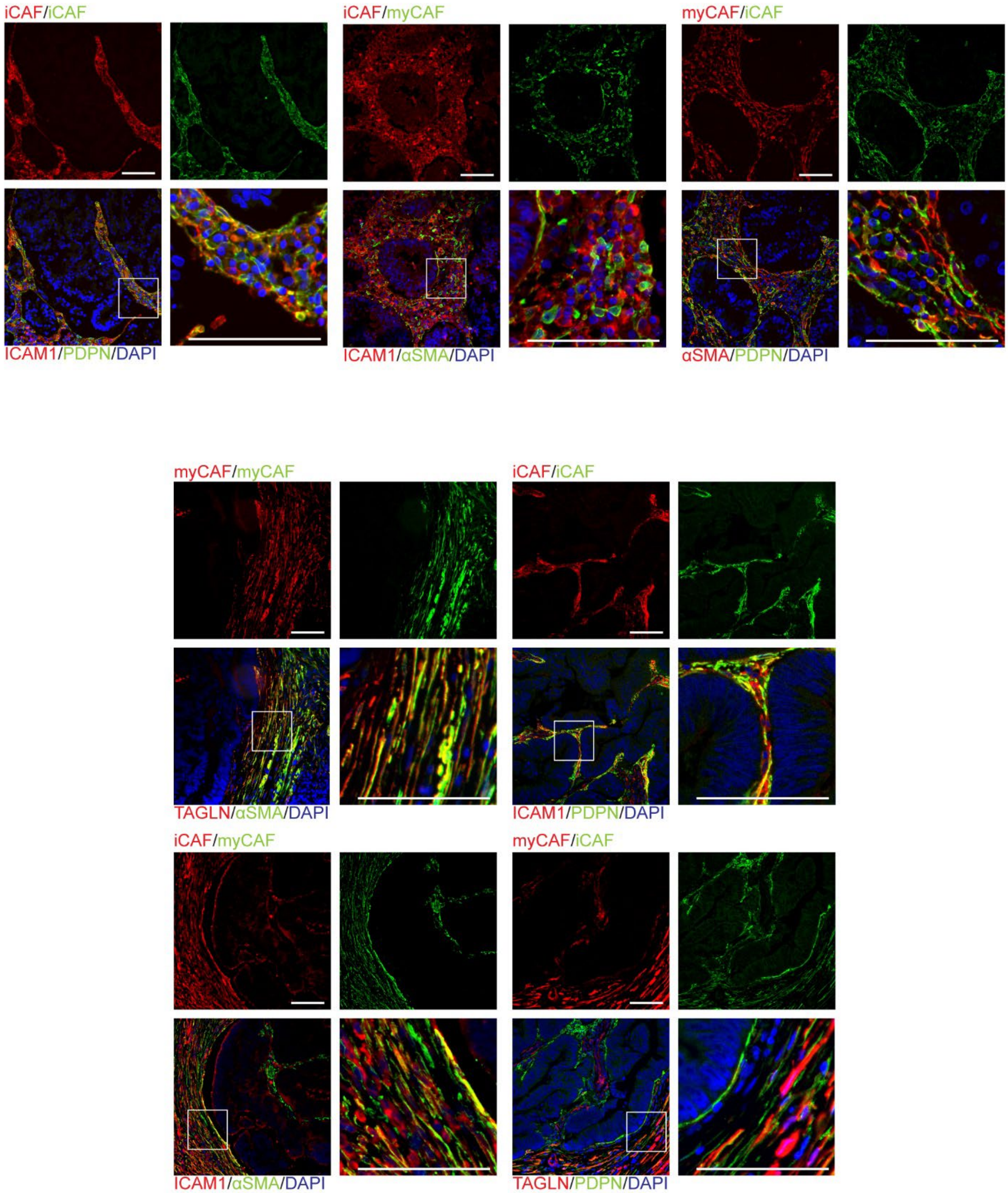

Fig. S1 cont'd

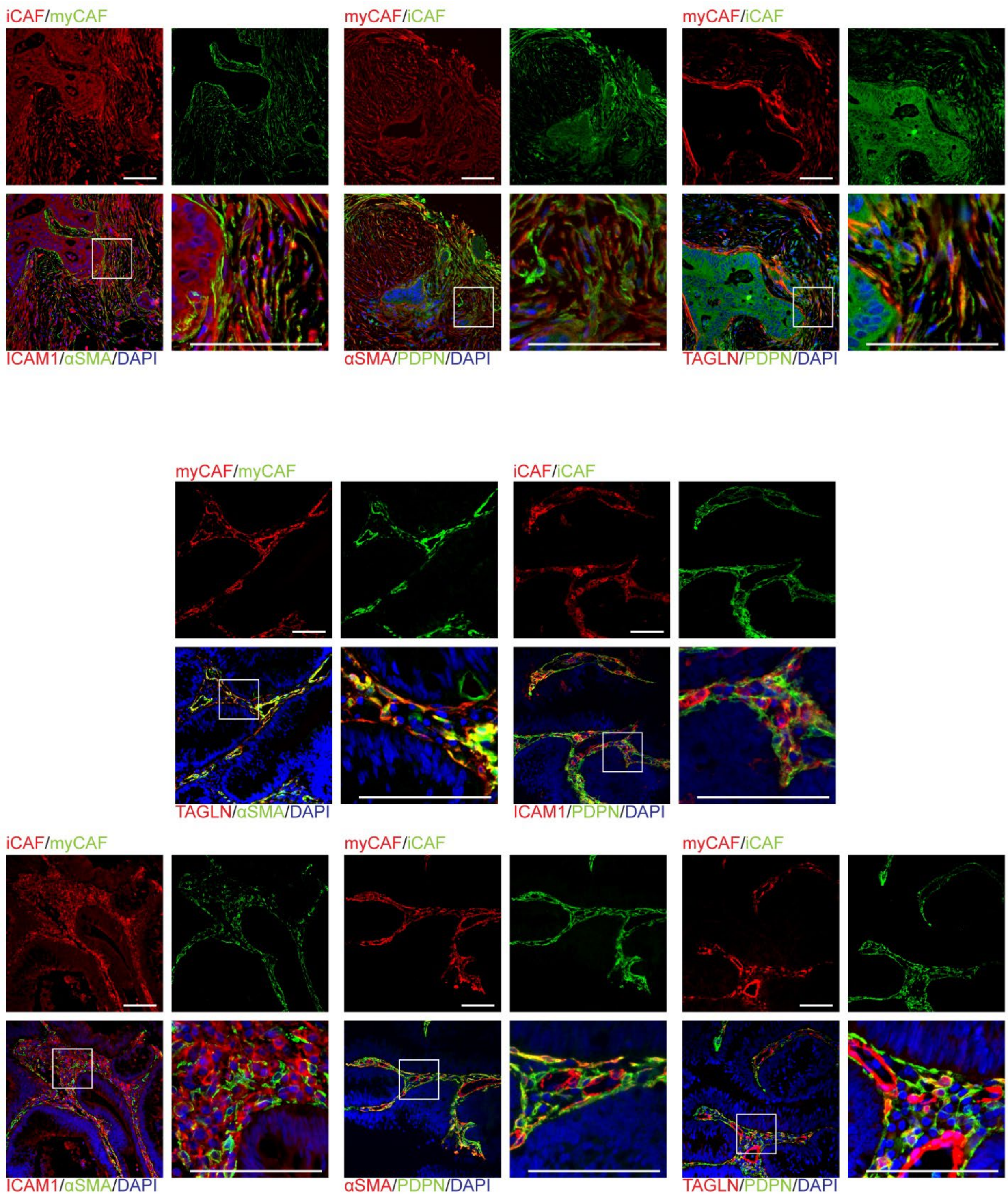

Fig. S1. Dual immunofluorescence of CAF markers across cancers. Each row represents one CRC patient sample. Scale bars 100 μM.

Fig. S2

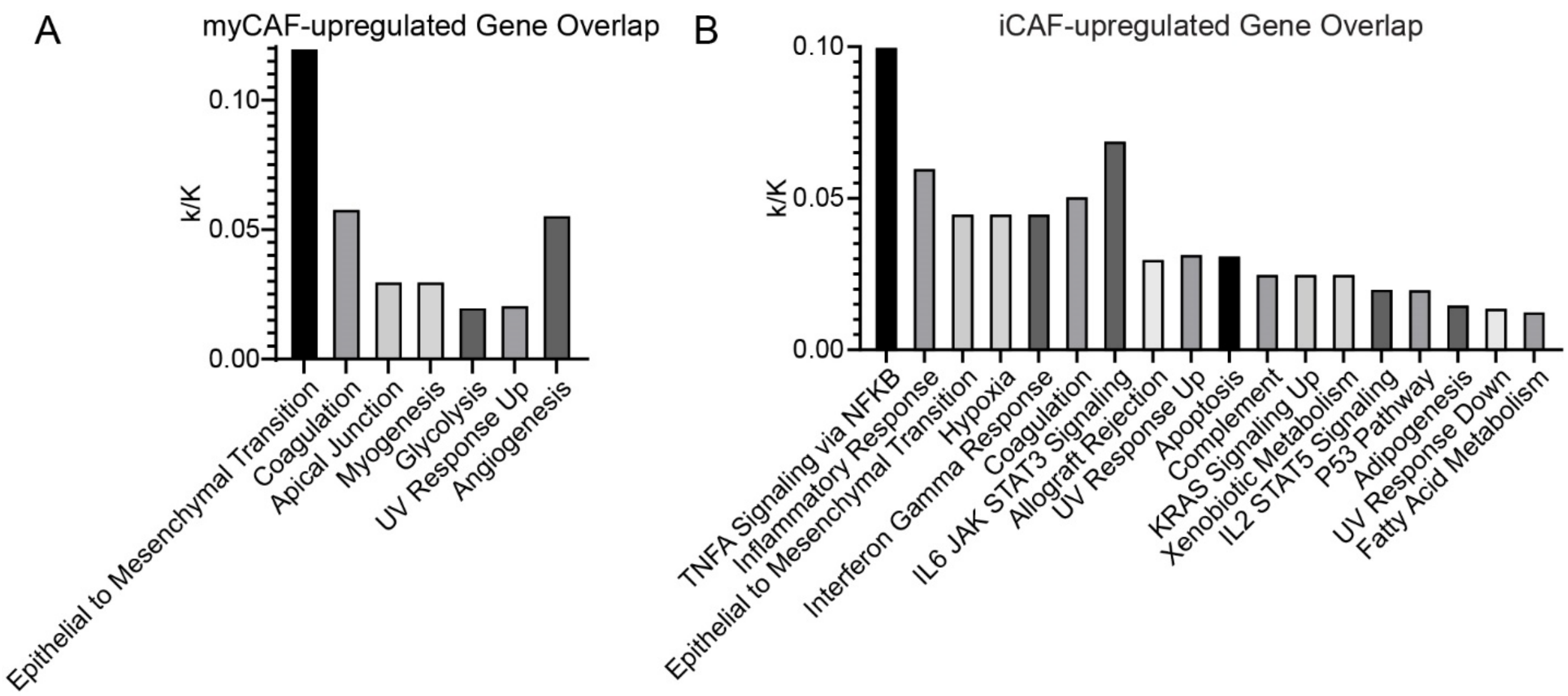

Fig. S2. Overlap of the top differentially expressed genes in the scRNA-seq dataset and hallmark gene sets. (A) Overlap of the top 50 genes upregulated in myCAFs versus iCAFs and the MSigDB hallmark gene sets. Presented as the % overlap, # of matching genes in query (k) / gene set size (K). (B) Overlap of the top 50 genes upregulated in iCAFs versus myCAFs.

Fig. S3

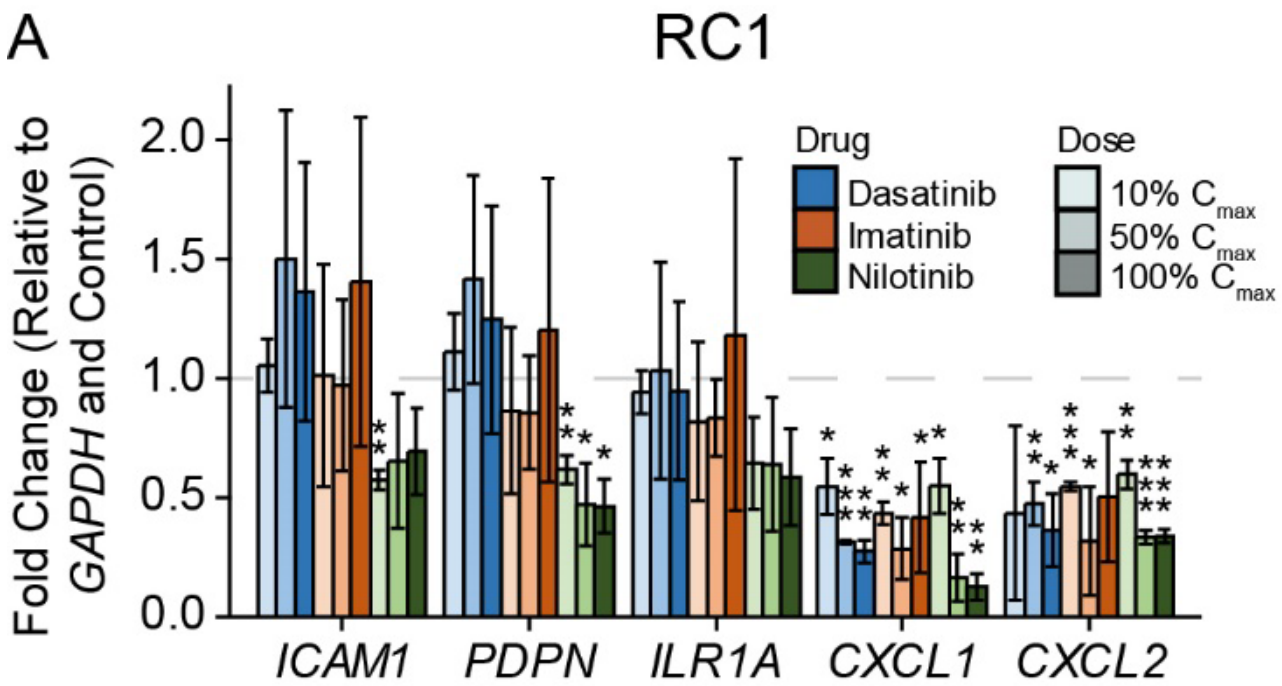

Fig. S3. Change in expression of iCAF genes with TKI treatment. Average change in expression of iCAF genes *ICAM1, PDPN, CXCL1, CXCL2,* and *IL1R1* over 96h in (A) RC1 and (B) RC2 cells treated with 10%, 50%, and 100% physiological C_{max} of nilotinib and related tyrosine kinase inhibitors imatinib and dasatinib measured by RT-qPCR. *$p < 0.05$, **$p < 0.01$, ***$p < 0.001$.

Fig. S4

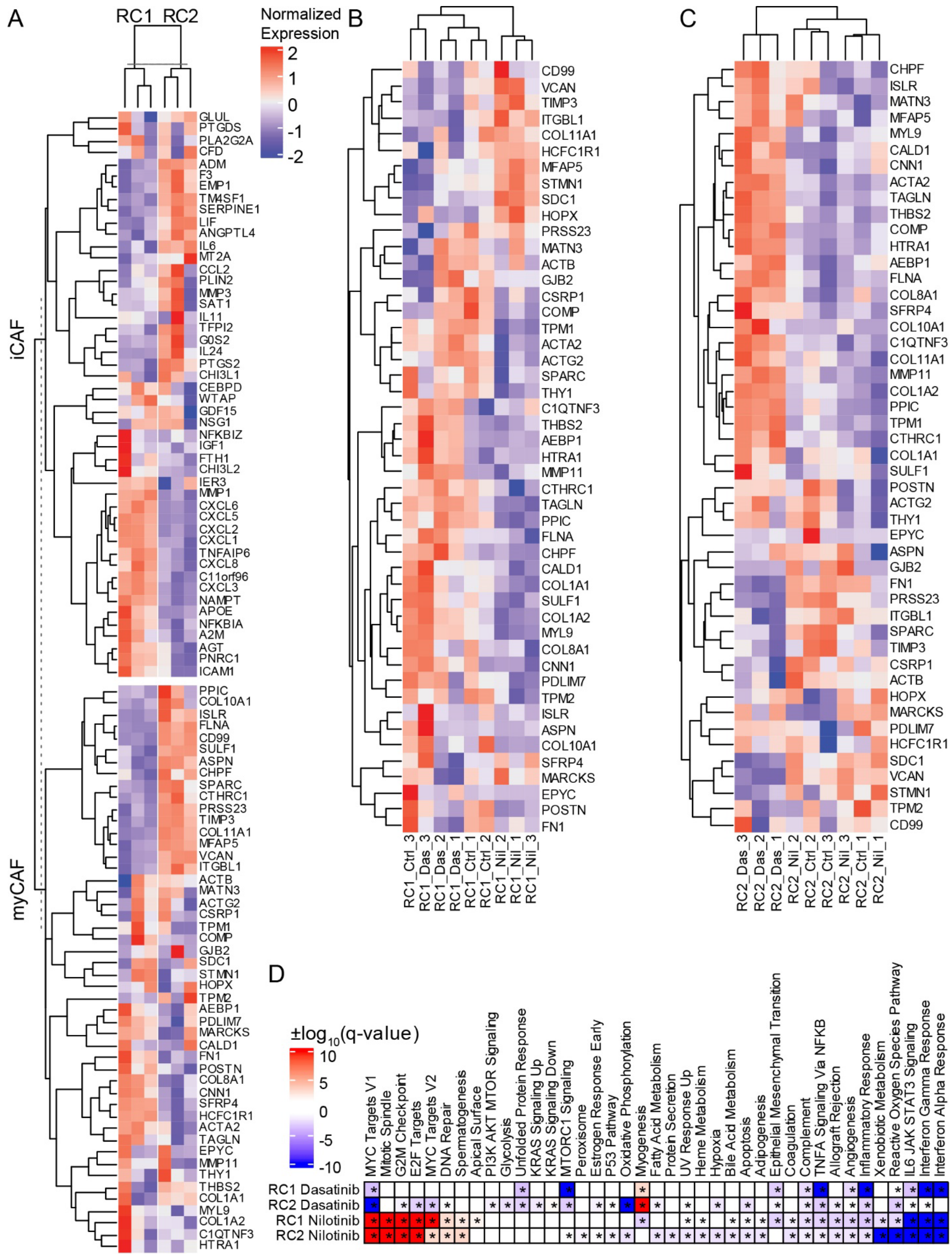
A
RC1 RC2
Normalized Expression
2
1
0
-1
-2
iCAF
myCAF
GLUL
PTGDS
PLA2G2A
CFD
ADM
F3
EMP1
TM4SF1
SERPINE1
LIF
ANGPTL4
IL6
MT2A
CCL2
PLIN2
MMP3
SAT1
IL11
TFPI2
G0S2
IL24
PTGS2
CHI3L1
CEBPD
WTAP
GDF15
NSG1
NFKBIZ
IGF1
FTH1
CHI3L2
IER3
MMP1
CXCL6
CXCL5
CXCL2
CXCL1
TNFAIP6
CXCL8
C11orf96
CXCL3
NAMPT
APOE
NFKBIA
A2M
AGT
PNRC1
ICAM1
PPIC
COL10A1
ISLR
FLNA
CD99
SULF1
ASPN
CHPF
SPARC
CTHRC1
PRSS23
TIMP3
COL11A1
MFAP5
VCAN
ITGBL1
ACTB
MATN3
ACTG2
CSRP1
TPM1
COMP
GJB2
SDC1
STMN1
HOPX
TPM2
AEBP1
PDLIM7
MARCKS
CALD1
FN1
POSTN
COL8A1
CNN1
SFRP4
HCFC1R1
ACTA2
TAGLN
EPYC
MMP11
THY1
THBS2
COL1A1
MYL9
COL1A2
C1QTNF3
HTRA1
B
CD99
VCAN
TIMP3
ITGBL1
COL11A1
HCFC1R1
MFAP5
STMN1
SDC1
HOPX
PRSS23
MATN3
ACTB
GJB2
CSRP1
COMP
TPM1
ACTA2
ACTG2
SPARC
THY1
C1QTNF3
THBS2
AEBP1
HTRA1
MMP11
CTHRC1
TAGLN
PPIC
FLNA
CHPF
CALD1
COL1A1
SULF1
COL1A2
MYL9
COL8A1
CNN1
PDLIM7
TPM2
ISLR
ASPN
COL10A1
SFRP4
MARCKS
EPYC
POSTN
FN1
RC1_Ctrl_3
RC1_Das_3
RC1_Das_2
RC1_Das_1
RC1_Ctrl_1
RC1_Ctrl_2
RC1_Nil_2
RC1_Nil_1
RC1_Nil_3
C
CHPF
ISLR
MATN3
MFAP5
MYL9
CALD1
CNN1
ACTA2
TAGLN
THBS2
COMP
HTRA1
AEBP1
FLNA
COL8A1
SFRP4
COL10A1
C1QTNF3
COL11A1
MMP11
COL1A2
PPIC
TPM1
CTHRC1
COL1A1
SULF1
POSTN
ACTG2
THY1
EPYC
ASPN
GJB2
FN1
PRSS23
ITGBL1
SPARC
TIMP3
CSRP1
ACTB
HOPX
MARCKS
PDLIM7
HCFC1R1
SDC1
VCAN
STMN1
TPM2
CD99
RC2_Das_3
RC2_Das_2
RC2_Das_1
RC2_Nil_2
RC2_Ctrl_2
RC2_Ctrl_3
RC2_Nil_3
RC2_Ctrl_1
RC2_Nil_1
D
±log10(q-value)
10
5
0
-5
-10
MYC Targets V1
Mitotic Spindle
G2M Checkpoint
E2F Targets
MYC Targets V2
DNA Repair
Spermatogenesis
Apical Surface
PI3K AKT MTOR Signaling
Glycolysis
Unfolded Protein Response
KRAS Signaling Up
KRAS Signaling Down
MTORC1 Signaling
Peroxisome
Estrogen Response Early
P53 Pathway
Oxidative Phosphorylation
Myogenesis
Fatty Acid Metabolism
Protein Secretion
UV Response Up
Heme Metabolism
Hypoxia
Bile Acid Metabolism
Apoptosis
Adipogenesis
Epithelial Mesenchymal Transition
Coagulation
Complement
TNFA Signaling Via NFKB
Allograft Rejection
Angiogenesis
Inflammatory Response
Xenobiotic Metabolism
Reactive Oxygen Species Pathway
IL6 JAK STAT3 Signaling
Interferon Gamma Response
Interferon Alpha Response
RC1 Dasatinib
RC2 Dasatinib
RC1 Nilotinib
RC2 Nilotinib

Fig. S4. RNA-seq shows differences between patient-derived CAF lines at baseline and in their response to nilotinib and dasatinib. (A) Expression of myCAF and iCAF genes across three biological replicates of RC1 and RC2. (B) Expression of myCAF genes upon treatment with dasatinib and nilotinib in RC1 CAFs. (C) Expression of myCAF genes upon treatment with dasatinib and nilotinib in RC2 CAFs. (D) GSEA results in RC1 and RC2 cells treated with dasatinib or nilotinib; $*q < 0.05$.

Fig. S5

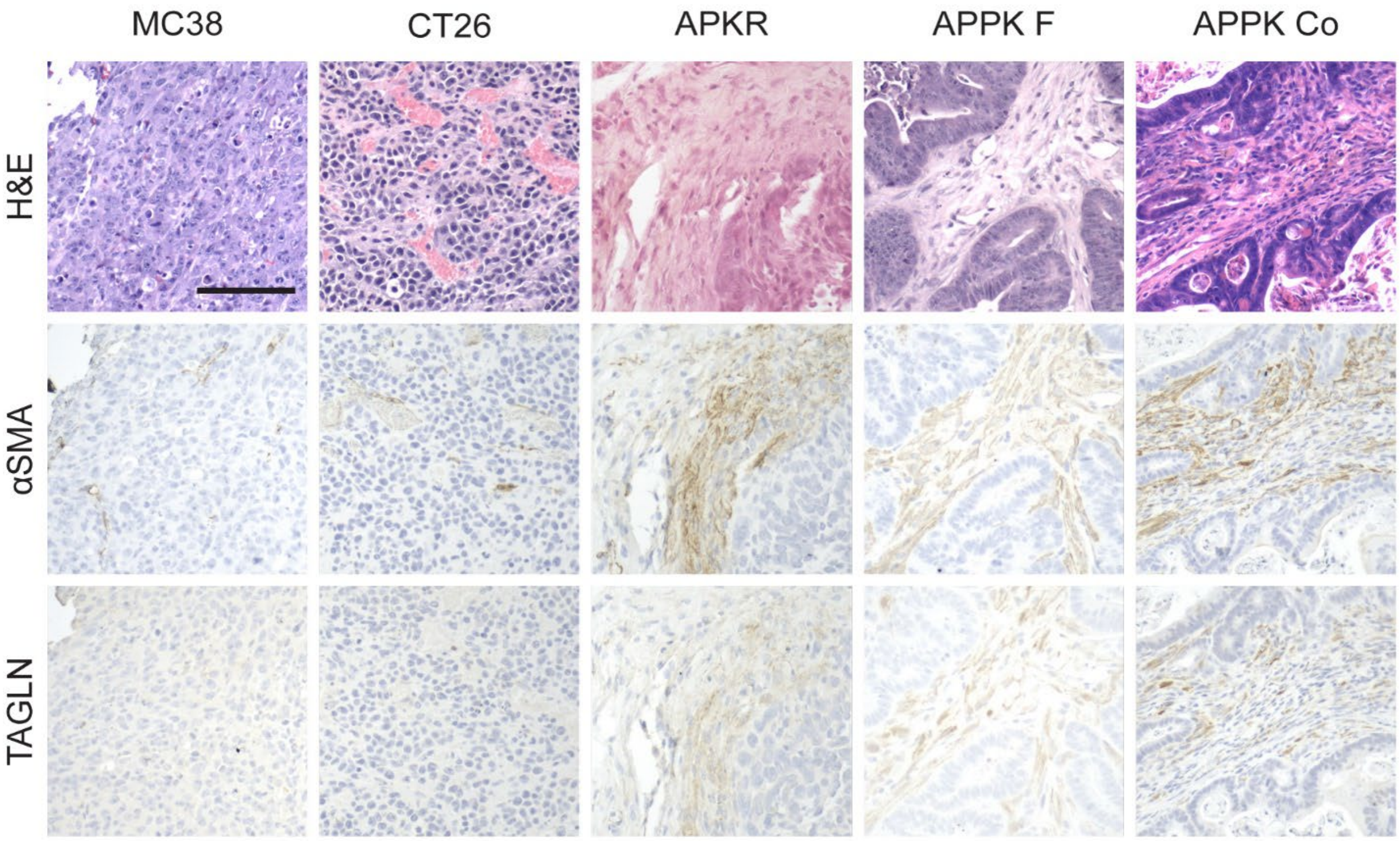

Fig. S5. Representative myCAF staining in various immunocompetent mouse models. MC38: MC38 flank-injected tumors. CT26: CT26 flank-injected tumors. APKR: *Apc*$^{fl/fl}$*Kras*^{G12D} genetic mice. APPK F: flank-injected APPK-derived organoid tumors. APPK Co: APPK-derived organoid, B6-derived normal colonic fibroblast flank co-injected tumors.

Fig. S6

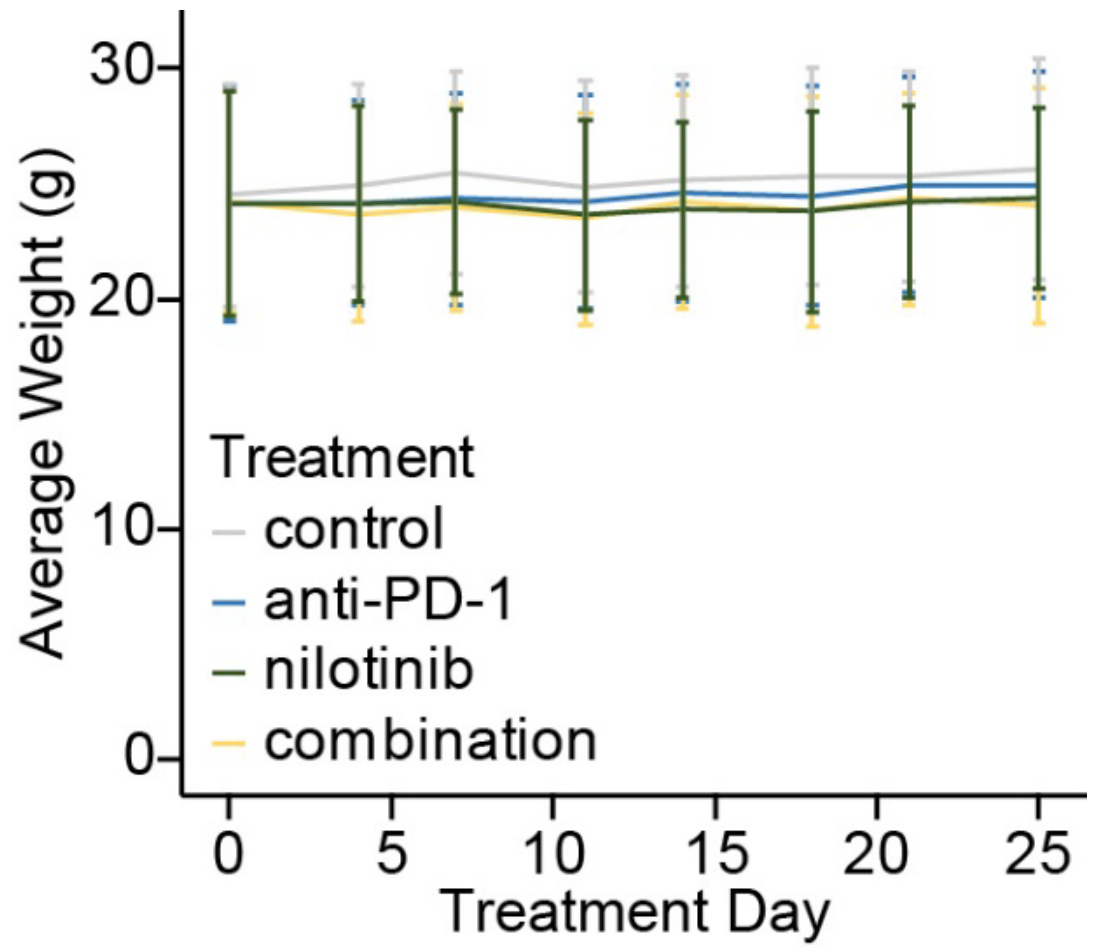

Fig. S6. Mouse weights do not change over the course of nilotinib and anti-PD-1 treatment. Average mouse weight over the 28-day treatment of combination nilotinib and anti-PD-1 treatment.

	myCAF High (N = 42)	iCAF High (N = 38)	Both High (N=79)	Neither High (N=62)
**Mean Age** (Range)	54.7 (32-87)	54.1 (25-86)	53.8 (13-90)	56.9 (22-90)
**Gender**				
Male (%)	23 (54.8)	27 (71.1)	58 (73.4)	35 (56.5)
Female (%)	19 (45.2)	11 (28.9)	21 (26.6)	27 (43.5)
**Stage**				
I (%)	8 (19.0)	14 (36.8)	20 (25.3)	16 (25.8)
II (%)	12 (28.6)	3 (7.9)	12 (15.2)	17 (27.4)
III (%)	14 (33.3)	13 (34.2)	29 (36.7)	13 (21.0)
IV (%)	8 (19.0)	8 (21.1)	18 (22.8)	16 (25.8)
**Disease Site**				
Left Colon (%)	32 (76.2)	22 (57.9)	53 (67.1)	34 (54.8)
Right Colon (%)	10 (23.8)	16 (42.1)	25 (31.6)	25 (40.3)
NA (%)	0 (0.0)	0 (0.0)	1 (1.3)	3 (4.8)
**Mismatch Repair**				
Deficient	7 (16.7)	7 (18.4)	10 (12.7)	12 (19.4)
Proficient	35 (83.3)	31 (81.6)	69 (87.3)	50 (80.6)

Table S1. Demographics of CAF subsets.

Gene	Forward	Reverse
GAPDH	5’-AAT CCC ATC ACC ATC TTC CA-3'	5’-TGG ACT CCA CGA CGT ACT CA-3'
ACTA2	5’-GGG ACG ACA TGG AAA AGAA TCT G-3'	5’-CAG GGT GGG AATG CTC TTC A-3'
TAGLN	5’-GGC AGC ACT GCA GAG GAC-3'	5’-TTA TGC TCC TGC GCT TTC TT-3'
COL11A1	5’-ACG CTG CAT ATA CAG GTA CCA TTT AG-3'	5’-TCA GCC CTG TTT CCA TCT TAG C-3'
ICAM1	5’-AGC GGC TGA CGT GTG CAG TAA T-3'	5’-TCT GAG ACC TCT GGC TTC GTC A-3'
PDPN	5’-AAC CAG CGA AGA CCG CTA TAA-3'	5’-CGA ATG CCT GTT AACA CTG TTG A-3'
CXCL1	5’-CCC AAA CCG AAG TCA TAG CCA-3'	5’-GAT GCA GGAA TTG AGG CAA GC-3'
CXCL2	5’-AGC TTG TCT CAA CCC CGC ATC-3'	5’-TTA GGC GCA ATC CAG GTG GC-3'
IL1R1	5’-ATG AAA TTG ATG TTC GTC CCT GT-3'	5’-ACC ACG CAA TAG TAA TGT CCT G-3'
Gapdh	IDT PrimeTime #Mm.PT.39a.1	
Acta2	5’-ACT GGG ACG ACA TGG AAA AG-3'	5’-GTT CAG TGG TGC CTC TGT CA-3'
Tagln	5’-CCT AGG CGG CCT TTA AAC C-3'	5’-CAT GCC GTA GGA TGG ACC C-3'
Col1a1	5’-CAT GGC CAA GAA GAC ATC CCT G-3’	5’-GCA TAC CTC GGG TTT CCA CG-3’
Col11a1	5’-CTT AAC ATC CAA GGC TGC CCA-3’	5’-TGC CTC TGT TTG TGC TAC TGT T-3’

Tgfb1	5'-TGA CGT CAC TGG AGT TGT ACG G-3'	5'-GGT TCA TGT CAT GGA TGG TGC-3'
Vegfa	5'-CCC GGG CCT CGG TTC-3'	5'-CCT GGG ACC ACT TGG CA-3'
Icam1	5'-ATG GGA ATG TCA CCA GGA ATG-3'	5'-TCA CGAA GGC CCA CAA TGA-3'
Cxcl2	5'-CCA ACC ACC AGG CTA CAG G-3'	5'-GCG TCA CAC TCA AGC TCT G-3'
Cxcl12	5'-GAG AAA GCT TTA AAC AAG GGC C-3'	5'-CGA GTT ACA AAG CGC CAG AG-3'
Myocd	5'-ACA CTC CTG GGG TCT GAA CA-3'	5'-GCG GTA TTA AGC CTT GGT TAG C-3'
Mrtfa	5'-GGT TCA GTG CCA TCA CCC TT-3'	5'-GGG AGT TGA GAA AGA ACC TGA GT-3'
Myh11	5'-GAA AGA CAG CAG CAT CAC GG-3'	5'-GCG AAT GAA CTT GCC AAA GC-3'
Cnn1	5'-TGC GCT TGT CTG TGT CAT CT-3'	5'-TCT GGG CCA GCT TGT TCT TT-3'

Table S2. Primers used in qPCR experiments.

Target	Company	Cat #	RRID	Clone	Concentration
αSMA	CST	19245	AB_2734735	D4K9N	1:250
TAGLN	Abcam	ab155272	AB_2637003	Polyclonal	1:1000
PDPN	CST	26981	AB_2798936	LpMab-12	1:250
ICAM1	CST	67836	AB_2799738	E3Q9N	1:250
MYOCD	Invitrogen	PA5-100775	AB_2850278	Polyclonal	1:1000
MRTFA	CST	97109	AB_3696707	E2V2I	1:100
CD8a (Human)	eBioScience	14-0085-80	AB_11151339	C8/144B	1:1000
Cd8a (Mouse)	CST	98941	AB_2756376	D4W2Z	1:200
GzmB	CST	44153	AB_2857976	E5V2L	1:100

Table S3. Antibodies and dilutions used in immunohistochemistry experiments.

Target	Company	Cat #	RRID	Clone	Antigen Presentation	Concentration
αSMA	CST	19245	AB_2734735	D4K9N	Citrate pH 6.0	1:250
αSMA	CST	56856	AB_2799522	1A4	Citrate pH 6.0	1:100
TAGLN	Abcam	ab155272	AB_2637003	Polyclonal	Citrate pH 6.0	1:1000
PDPN	CST	26981	AB_2798936	LpMab-12	Citrate pH 6.0	1:50
ICAM1	CST	67836	AB_2799738	E3Q9N	Citrate pH 6.0	1:50
CD4	Thermo-Fisher	14-2444-82	AB_2572868	N1UG0	EDTA pH 9.0	1:100
FOXP3	CST	98377	AB_2747370	D2W8E	EDTA pH 9.0	1:100
TBET	CST	13232	AB_2616022	D6N8B	EDTA pH 9.0	1:1000

Table S4. Antibodies and dilutions used in immunofluorescence experiments.
